# Balancing selection on a recessive lethal deletion with pleiotropic effects on two neighboring genes in the porcine genome

**DOI:** 10.1371/journal.pgen.1007661

**Published:** 2018-09-19

**Authors:** Martijn F. L. Derks, Marcos S. Lopes, Mirte Bosse, Ole Madsen, Bert Dibbits, Barbara Harlizius, Martien A. M. Groenen, Hendrik-Jan Megens

**Affiliations:** 1 Wageningen University & Research, Animal Breeding and Genomics, Wageningen, The Netherlands; 2 Topigs Norsvin Research Center, Beuningen, the Netherlands; 3 Topigs Norsvin, Curitiba, Brazil; University of Bern, SWITZERLAND

## Abstract

Livestock populations can be used to study recessive defects caused by deleterious alleles. The frequency of deleterious alleles including recessive lethal alleles can stay at high or moderate frequency within a population, especially if recessive lethal alleles exhibit an advantage for favourable traits in heterozygotes. In this study, we report such a recessive lethal deletion of 212kb (del) within the *BBS9* gene in a breeding population of pigs. The deletion produces a truncated BBS9 protein expected to cause a complete loss-of-function, and we find a reduction of approximately 20% on the total number of piglets born from carrier by carrier matings. Homozygous del/del animals die mid- to late-gestation, as observed from high increase in numbers of mummified piglets resulting from carrier-by-carrier crosses. The moderate 10.8% carrier frequency (5.4% allele frequency) in this pig population suggests an advantage on a favourable trait in heterozygotes. Indeed, heterozygous carriers exhibit increased growth rate, an important selection trait in pig breeding. Increased growth and appetite together with a lower birth weight for carriers of the *BBS9* null allele in pigs is analogous to the phenotype described in human and mouse for (naturally occurring) *BBS9* null-mutants. We show that fetal death, however, is induced by reduced expression of the downstream *BMPER* gene, an essential gene for normal foetal development. In conclusion, this study describes a lethal 212kb deletion with pleiotropic effects on two different genes, one resulting in fetal death in homozygous state (*BMPER*), and the other increasing growth (*BBS9*) in heterozygous state. We provide strong evidence for balancing selection resulting in an unexpected high frequency of a lethal allele in the population. This study shows that the large amounts of genomic and phenotypic data routinely generated in modern commercial breeding programs deliver a powerful tool to monitor and control lethal alleles much more efficiently.

## Author summary

We report a large deletion within the *BBS9* gene that induces late fetal mortality in homozygous affected animals in a commercial pig population. This late fetal mortality causes the fetus to become encapsulated and desiccated during the remaining time of the pregnancy, a process called mummification. The unusually high carrier frequency for this lethal deletion (10.8%) likely results from its strong positive association with growth rate in heterozygous individuals, an important selection trait in the pig breeding industry. Interestingly, we show that the positive effect on growth is induced by a heterozygous loss-of-function of the *BBS9* gene, associated with obesity in human and mouse. However, late fetal mortality is induced by insufficient expression of the *BMPER* gene located directly downstream of the deletion which affects its regulatory elements required for gene expression. Together, our study shows an unique example of allelic pleiotropy in which one allele (deletion) is responsible for both increased growth and late fetal mortality by affecting two different genes.

## Introduction

Domesticated animals are excellent models to study the effect of inbreeding on fitness, and the role of selection in inbreeding depression. Breeding of domesticated animals increases inbreeding by applying artificial insemination that allows breeding populations to be sired by a small number of elite males. The frequency of deleterious alleles including recessive lethal alleles can rise in populations as a consequence of drift due to small effective population size, but also due to selection [1]. Inherited defects usually derive from unique “founder” mutations [2]. Especially in cattle breeds, several high frequency lethal alleles have been described [3, 4] reaching carrier frequencies up to 32% [5], that can be traced back to prime bulls that were used extensively in the past decades. However, the effect of individual sires on the population depends on the breeding goal and the structure of the breeding program. In cattle breeding, the genetic contribution of a single bull can be extreme, producing up to hundreds of thousands of daughters. In pig breeding, however, drift effects are expected to be less severe because recessive lethal alleles from founder boars are less likely to rise in frequency very rapidly, because of a lower male selection intensity compared to cattle breeding [6].

The role of random drift and/or selection in increasing the frequency of deleterious variants is complex. When effective population size is small, drift effects can result in less effective selection [7]. Interestingly, the number of lethal variants found at relatively high frequency in commercial pig populations appears to be low [8–10]. The relative paucity of high-frequency deleterious alleles in pig and chicken, species that generally show a more gender-balanced selection [6, 11], and larger effective population size compared to cattle breeds [12, 13], raises the question why still some alleles rise to moderate frequency despite having a very clear adverse effect. Heterozygote advantage for traits selected in commercial populations provides a tantalizing alternative hypothesis [14]. In cattle, various instances of balancing selection have been described, driving deleterious alleles to higher population frequencies [5, 15]. In pigs, similar observations were made involving a transposable element (L1) insertion with positive effect on litter size, but negative consequences for boar fertility [16].

In a previous study we identified various recessive lethal alleles in three pig breeds [8], but the majority of these lethal alleles were found at low frequencies. One recessive lethal haplotype, however, was found at moderate frequency (~9% carrier frequency) causing a significant increase in foetal mortality at mid- to late-gestation and resulting in a high fraction of mummified piglets in a Large White commercial population. The strong deleterious nature of the allele and the high frequency suggests a factor other than drift driving this haplotype to high frequency.

In this study, we report evidence of balancing selection on a recessive lethal 212kb deletion within the *BBS9* gene with antagonistic effects on fertility and growth. The allele affects fertility by causing early fetal death in homozygous progeny, resulting in mummified piglets. The same allele increases growth rate and feed intake for carrier animals compared to non-carrier animals. We propose that the deletion is maintained at moderate frequency in the Large White breed because of its association with this positive effect, despite it being lethal in homozygous state.

## Results

### A haplotype inducing foetal lethality segregates at moderate frequency in a Large White pig population

Genomic loci that harbour recessive lethal alleles can be identified by searching for haplotypes showing reduced or missing homozygosity. In this study, we analysed a previously identified recessive lethal haplotype on pig chromosome 18 (SSC18: 39.25–40.1 Mb) using 23,722 Large White animals from a single purebred sow line genotyped on the Porcine50K SNPchip (Sscrofa11.1 build). The haplotype frequency is estimated at 5.4% (10.8% carrier frequency, Table 1), showing that the haplotype is segregating at moderate frequency in this Large White population. In total, we expect 55 homozygote carriers for the SSC18 haplotype within the population. However, no homozygous del/del animals were observed, supporting that all copies of the haplotype carry the recessive lethal variant exhibiting complete penetrance for homozygous animals. We also observe a significant reduction in total number born (19.5%) and liveborn individuals (19.3%) for carrier-by-carrier matings (CxC) compared to carrier-by-non-carrier matings (CxNC). Moreover, we found an approximate fivefold increase in mummified piglets (Table 1). The difference between stillborn and mummies lies in the moment the foetus dies: The term ‘mummy’ is used for a foetus that dies mid-to-late-gestation (e.g. second to third trimester) and is subsequently encapsulated and desiccated during the remainder time of the pregnancy. A foetus that dies near the end of gestation or perinatally is identified as ‘stillborn’. The reduction in total number born is slightly lower than the expected 25% based on the 1:2:1 genotype distribution expected from CxC matings. About 73% of the CxC progeny is heterozygous for the SSC18 haplotype, corresponding to the 1:2 genotype ratio expected for CxC matings that lack homozygous offspring, significantly different compared to the normal 1:2:1 Mendelian ratio (p = <0.00001). Based on the carrier frequency, we estimate that about 1.17% of the litters within this breed are affected by the SSC18 haplotype, producing affected animals (‘mummies’), and resulting in reduced litter sizes (on average 3.08 piglets per CxC litter).

**Table 1:** SSC18 haplotype characteristics and phenotypic effects. Difference is the percent difference in the average total number born (TNB), number born alive (NBA), and mummified piglets (MUM) for C x C (carrier-by-carrier) and C x NC (carrier-by-non-carrier) matings.

**Position, Mb**	SSC18: 39.2–40.1
**Number of markers**	25
**Starting marker**	ASGA0079708
**Ending marker**	ALGA0098146
**Homozygotes expected (trio)**	55
**Homozygotes observed**	0
**Exact binomial test**	1.12e-27
**Haplotype frequency %**	5.42
**Carrier frequency %**	10.84
**C x C matings**	154
**Genotyped C x C progeny**	218
**Heterozygote C x C progeny**	159 (72.9%)
**Avg. TNB (difference %)**	12.86 (-19.5%)
**Avg. NBA (difference %)**	11.69 (-19.3%)
**Avg. MUM (difference %)**	1.62 (476.4%)
**Genes in window**	BMPER, BBS9

### Genotyping the offspring of carrier-by-carrier matings confirms early lethality of homozygous animals

We tracked five recent CxC matings. Four pregnancies reached full term, while one resulted in spontaneous early abortion of the entire litter (Table 2). The four full-term litters produced 49 liveborn, 7 stillborn, and 14 mummified piglets. Each of these four litters produced at least 2 mummified piglets (maximum 5), significantly more than what is normally observed in this breed (on average 0.35 mummified piglets per litter, *p* = 0.0027). Among the total of 48 genotyped liveborn and stillborn siblings (8 siblings were not genotyped), 16 were non-carriers, 30 were heterozygous (62.5%), and two were homozygous for the SSC18 haplotype, close to the expected 1:2 genotype ratio caused by missing homozygous offspring (S1 and S2 Tables). Among the two "fresh born" homozygous animals (i.e. piglets surviving at least until around birth), one was a stillborn piglet, the other was a liveborn but very weak piglet, that died shortly after birth.

**Table 2:** Tracked CxC matings for the SSC18 haplotype. Phenotypes and genotypes of 4 litters from CxC matings from two different farms. The number of successfully genotyped individuals are indicated between parentheses for each birth type. Litter CC3 contains two fresh born homozygous individuals. An overview presenting the haplotypes and carrier status of the four litters is provided in S3 and S4 Tables.

Litter	Farm	Parity	Liveborn	Stillborn	Mummified	# Non-carriers	# Carriers	# Confirmed homozygotes
CC1	1	5	10 (6)	1 (1)	4 (0)	2	5	-
CC2	2	1	12 (11)	0	3 (1)	3	8	1
CC3	1	5	17 (15)	3 (3)	2 (0)	7	9	2
CC4	2	3	10 (10)	3 (2)	5 (1)	4	8	1

We confirmed the homozygous status for two mummified piglets with sufficient call rate (call rate > 0.8, S1 Table), the other mummified piglets yielded insufficient DNA quality to perform genotyping and phasing (call rate < 0.8, S1 and S2 Tables). Next, we collected eight mummified piglets from one farm for phenotypic evaluation (including X-rays, S1 Fig), the other six mummified piglets were measured (length), but not stored. The approximate age when a mummified pig has died can be determined based on the length (crown to rump) and weight. The majority of the mummified piglets die approximately in the second half of the second trimester of pregnancy (50–70 days), based on the length (100–200 mm) and weight (100–190 gram) of the mummified piglets (S1 and S2 Tables, S1 Fig). Three mummified piglets from one litter (litter ID: CC4) died later in gestation as was evident from a larger size and weight (S1 Table). However, we cannot confirm the homozygous status for the SSC18 haplotype, since these animals could not be successfully genotyped due to poor DNA quality. Together these results support a broad range in the time of death between homozygous animals (supporting variation in penetrance), ranging from 50 days in gestation to 24 hours post-partum.

### Carriers exhibit a 212kb deletion affecting the *BBS9* gene

**Fig 1:**
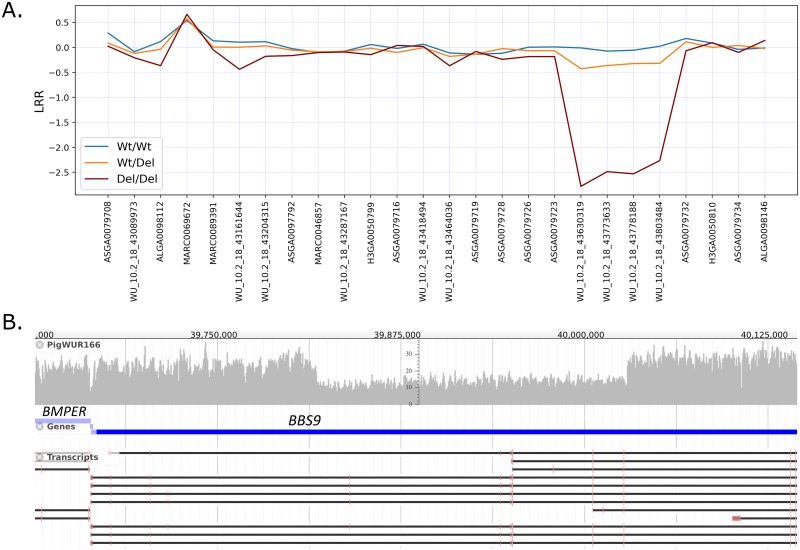
**A) Log R Ratio (LRR) signal intensities on the 50K SNPchip for homozygotes (del/del) carriers (wt/del), and non-carriers (wt/wt).** Four markers within the 212kb deletion show reduced LRR intensities. **B) Screen capture of the alignment of carrier animal PigWUR166.** The aligned region on SSC18 shows reduced coverage in the deletion within the *BBS9* gene.

To identify candidate causal mutations, we analysed whole genome sequence data from 73 individuals from the same Large White population and identified 10 carrier animals for the SSC18 haplotype (S5 Table). We first annotated loss-of-function and (deleterious) missense mutations within and surrounding the haplotype region (+/- 5 Mb) uniquely found in the SSC18 haplotype carriers. However, none of the mutations were predicted to have high impact (Variant Effect Predictor, build 90 [17]). Next, we assessed the presence of structural variation within the same region and identified a large deletion in complete LD with the SSC18 haplotype of approximately 212kb (position 39,817,373 to 40,029,300), spanning a part of the *BBS9* gene (Fig 1A and 1B). The deletion is supported by both split-reads and discordantly mapped pairs in carrier samples (S2 Fig). Moreover, carrier animals show reduced signal intensities (referred to as Log R Ratio; Fig 1A, S3 Fig), and increased homozygosity for four markers on the Porcine50K SNPchip located within deletion, caused by the absence of a second haplotype for the deletion region. In addition, several markers neighbouring the deletion show an excess of heterozygosity, caused by the absence of homozygous del/del animals.

### SSC18 deletion produces a truncated BBS9 protein

**Fig 2:**
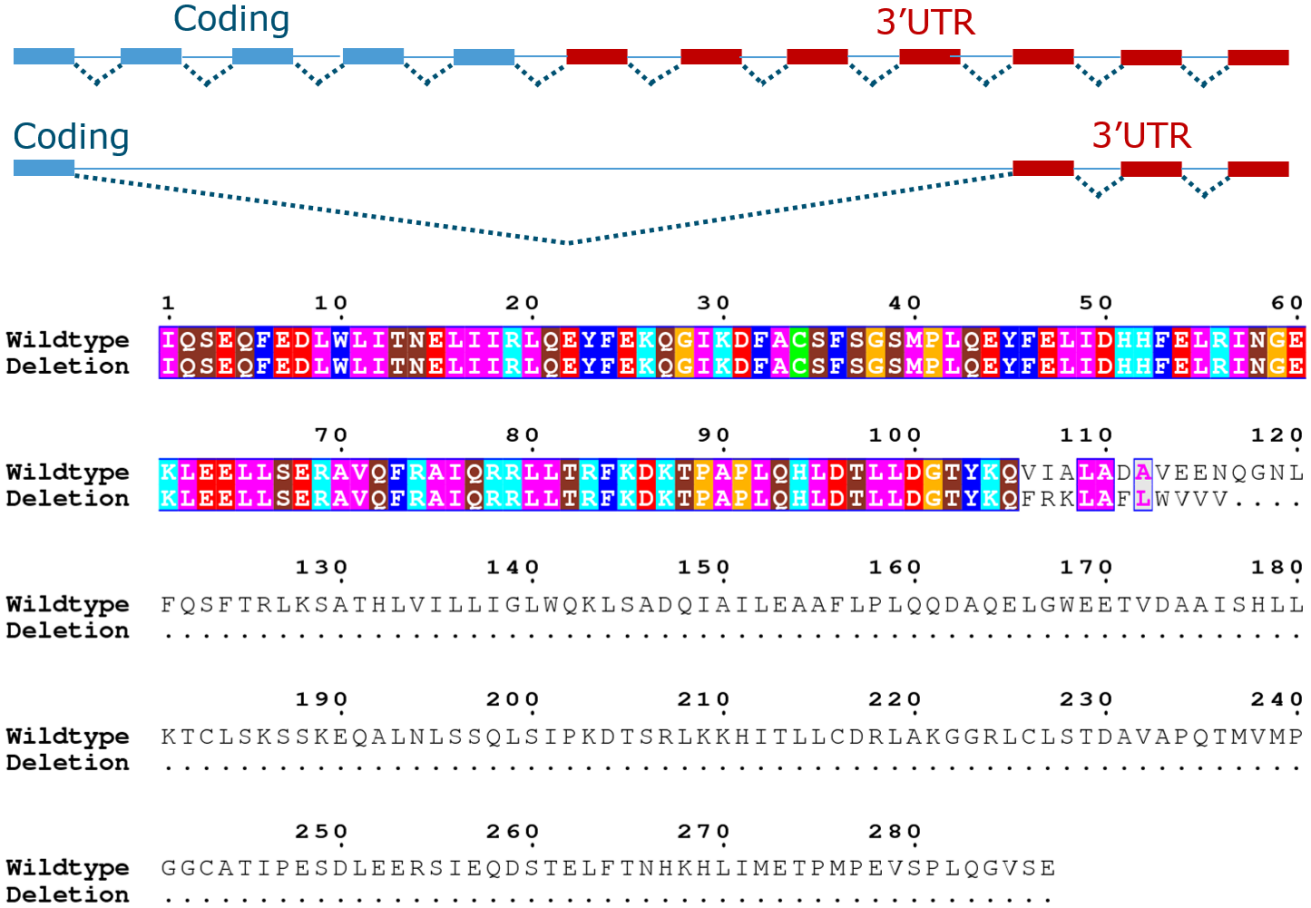
BBS9 “Wild-type” (top) and mutant (bottom) transcripts. The deletion transcript skips four coding and four 3’UTR exons, resulting in a frameshift (indicated with an arrow in the alignment) introducing 11 AAs before a preliminary stop codon.

We analysed RNA-seq data from one carrier animal in eight different tissue types (sample: PigWur166, S6 Table) to investigate the impact of the deletion on the expression of *BBS9*. Moderate gene expression levels for *BBS9* were observed for the majority of the examined tissues, except for muscle, and with highest gene expression in testis (S6 Table). We evaluated the effect of the deletion on the *BBS9* mRNA and show that the deletion induces skipping of 4 coding, and 4 3'UTR exons for the *BBS9* canonical transcript (Fig 2, RefSeq ID: XM_021079336.1), resulting in direct splicing from exon 19 to exon 28 (3'UTR). The mutant transcript results in a frameshift introducing 11 novel amino acids before a premature stop codon, generating a truncated *BBS9* protein of 694 amino acids (including 11 novel amino acids) instead of the wild type 865 amino acids. This truncated BBS9 protein will likely be non-functional (Fig 2), supported by pathogenic mutations identified in humans affecting the same C-terminal tail of the BBS9 protein [18, 19]. Moreover, the affected protein coding exons exhibit a negative subRVIS score, indicating intolerance to loss-of-function mutations [20]. Finally, we evaluated the expression of *BBS9* using a RT-qPCR on 8 carrier and 10 non-carrier samples from whole blood using primers that target exons located within the deletion. The results show a 50% lower expression of the wild-type *BBS9* gene in carrier animals (S4 Fig).

### SSC18 deletion lowers *BMPER* expression by affecting cis-regulatory elements

To evaluate the impact of the deletion on the downstream *BMPER* gene we investigated possible allelic imbalance for the *BMPER* gene within the same carrier animal. The *BMPER* gene is highly expressed in lung, while moderately expressed in the other tissue types (S6 Table). One heterozygous coding synonymous mutation within the fourth exon of the *BMPER* canonical transcript (XM_013990842.2) was used to test for allelic imbalance. Interestingly, we observed a three-fold higher expression of the *BMPER* allele for the wild-type haplotype (T allele) compared to the del haplotype (in lung tissue, Table 3). By contrast, three homozygous wild-type animals showed no allele specific differences in expression for the *BMPER* gene (S7 Table), suggesting that the region affected by the 212kb deletion contains *BMPER* cis-regulatory elements. To support the presence of *BMPER* regulatory elements within the deletion we aligned liver ChipSeq (H3K27Ac, H3K4Me3) data [21] to the Sscrofa11.1 genome build. Two strong enhancer peaks are observed within the deletion region, while only weak signals are observed outside the deletion region (S5 Fig). In addition, the sequence of the 212kb deletion was mapped to the human genome to identify the homologous sequence on the human genome (GRCh38: Chr7:33.50–33.71). This region contains several conserved regulatory elements, identified from the Regulatory Element Database [22], one non-coding RNA (LOC105375227), and several enhancer sites, of which at least two are annotated to enhance *BMPER* expression according to the human EnhancerAtlas [23].

**Table 3:** Allele specific expression of the *BMPER* gene for a SSC18 carrier animal. One heterozygous coding synonymous SNP within the fourth exon of the *BMPER* canonical transcript (XM_013990842.2) was used to test for allelic imbalance.

Locus	Gene	Del-allele	Alt-allele	Del- Count	Wt- Count	Ratio	FDR-p
18:39594479	*BMPER*	C	T	24	73	0.753	2.35e^-05^

### Tracing the origin of the deletion

**Fig 3:**
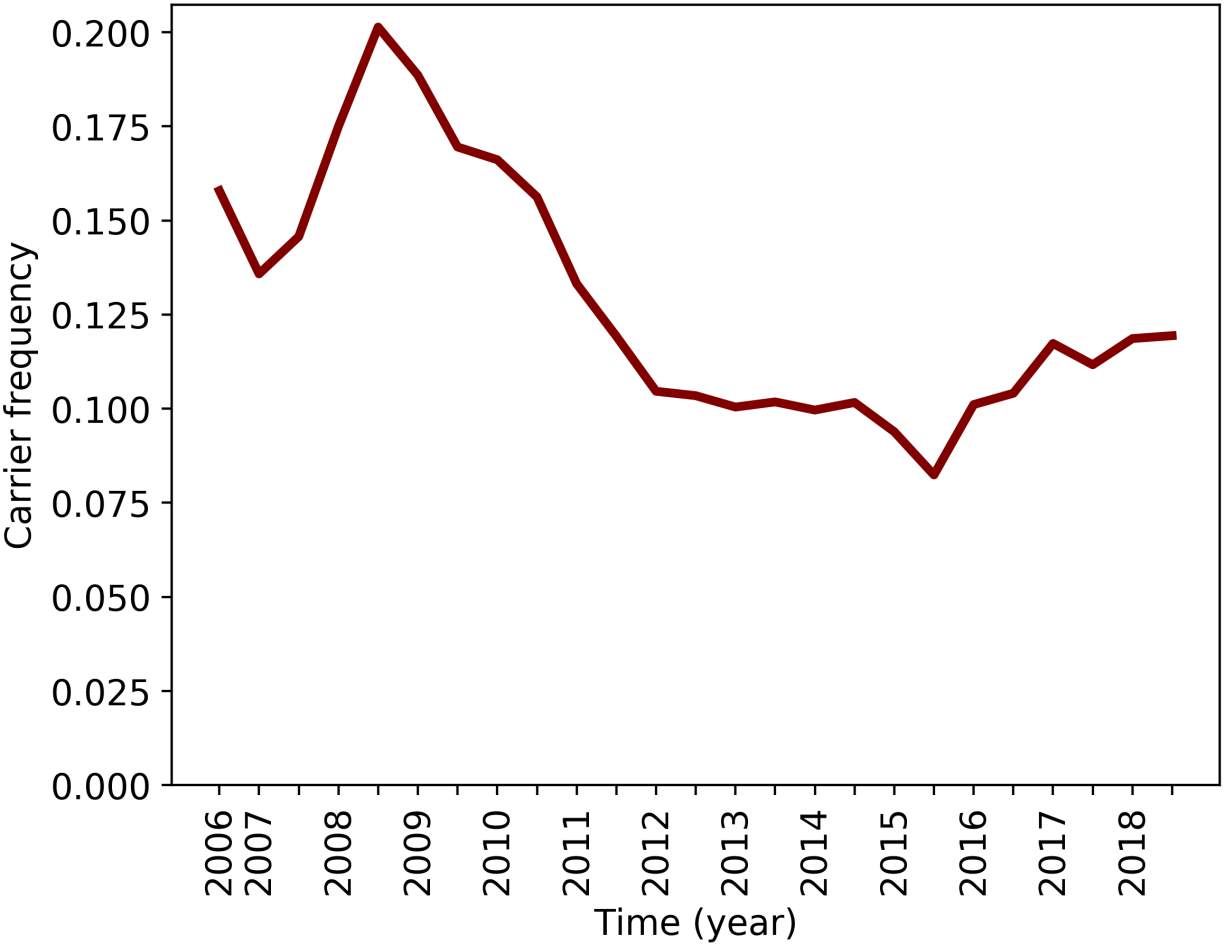
SSC18 carrier frequency from 2006–2018. The frequency has changed significantly over the past 12 years (p = 0.012).

To investigate the origin of the deletion, we analysed the frequency of the deletion over the last decades. The first born animals within our genotyped set are from February 2006, allowing the tracking of the frequency of the deletion over the past decade. The number of genotyped animals was lower in the period 2006–2010. However, we genotyped over 320 animals in the (live) population from 2008 onwards, providing reliable frequency estimates (S8 Table). The SSC18 haplotype carrier frequency was high (>15%) over the period 2006–2010 (maximum 20% in 2008) and then decreased to a relative stable ~10% carrier frequency from 2012 onwards (Fig 3).

The Large White population under study has been created out of the consolidation of a number of Dutch breeding organizations around the turn of the last century [24]. During the consolidation phase, which resulted in merging of populations and phasing out of other populations, sperm of breeding boars was deposited at the Dutch Centre for Genetic Resources (CGN). The current Large White pure line descends from two different populations, the StamBoek-Z and the Dumeco-W line [24]. Both breeds were merged around 2003 to form the current Large White breeding line. From the 11 StamBoek-Z boars available at CGN, none were carrier of the deletion. However, from the 56 genotyped Dumeco-W boars available at CGN, five were carrier for the deletion haplotype (8.9%). These boars were born in 2000 and 2001 (S9 Table), showing that the deletion derives from this ancestral line and has been maintained in the Large White population for the past eighteen years (~ 15 generations).

### Carriers of the deletion have increased growth rate and feed intake

We examined whether the current carrier frequency is purely the result of genetic drift, or whether carriers exhibit selective advantage for important traits within the breeding program. We first simulated genetic drift for a lethal recessive allele in the current Large White population (S7 Fig). The results show that lethal alleles can reach allele frequencies up to about 10% by drift alone (although extremely rare), at which the lack of homozygotes is preventing further increase. Next, we tested whether deletion carriers exhibit heterozygote advantage, by performing an association study for both carrier and non-carrier animals using deregressed estimated breeding values (DEBVs) for 16 production traits available from the Topigs-Norsvin breeding program (Table 4).

**Table 4:** Traits significantly associated with heterozygous carriers of the deletion. Effect shows the direction of the association, se shows the standard error. Table shows increased DEBVs for growth rate (TGR: growth rate in test period, ~25-120Kg, LGR: lifetime growth rate), daily feed intake (DFI), and litter mortality (LMO), while decreased DEBV for litter birth weight (LBW, grams), loin depth (LDE), and longevity (LGY) are observed for carriers. The symbols "+" and "-" indicate positive and negative effects. The effect on DFI can be considered both positive and negative. If TGR is increasing, DFI tends to increase a bit. However, it should not increase too much because it will affect feed conversion. An overview of all traits tested is provided in S10 Table.

Trait (unit)	Non-carriers	Carriers	P	-log10(P)	effect	se
TGR (gr/day) ^+^	15013	1605	0.000046	4.34	11.46	2.81
LDE (mm) ^¯^	15011	1598	0.000198	3.70	-0.45	0.12
LGR (gr/day) ^+^	15116	1616	0.000315	3.50	6.40	1.77
LBW (gram) ^¯^	6945	824	0.001232	2.91	-16.67	5.16
LMO (%) ^¯^	7345	871	0.001248	2.90	0.67	0.21
DFI (gr/day) ^+/-^	14671	1567	0.006764	2.17	30.61	11.30
LGY (parity) ^¯^	7250	856	0.024828	1.61	-0.08	0.04

**Fig 4:**
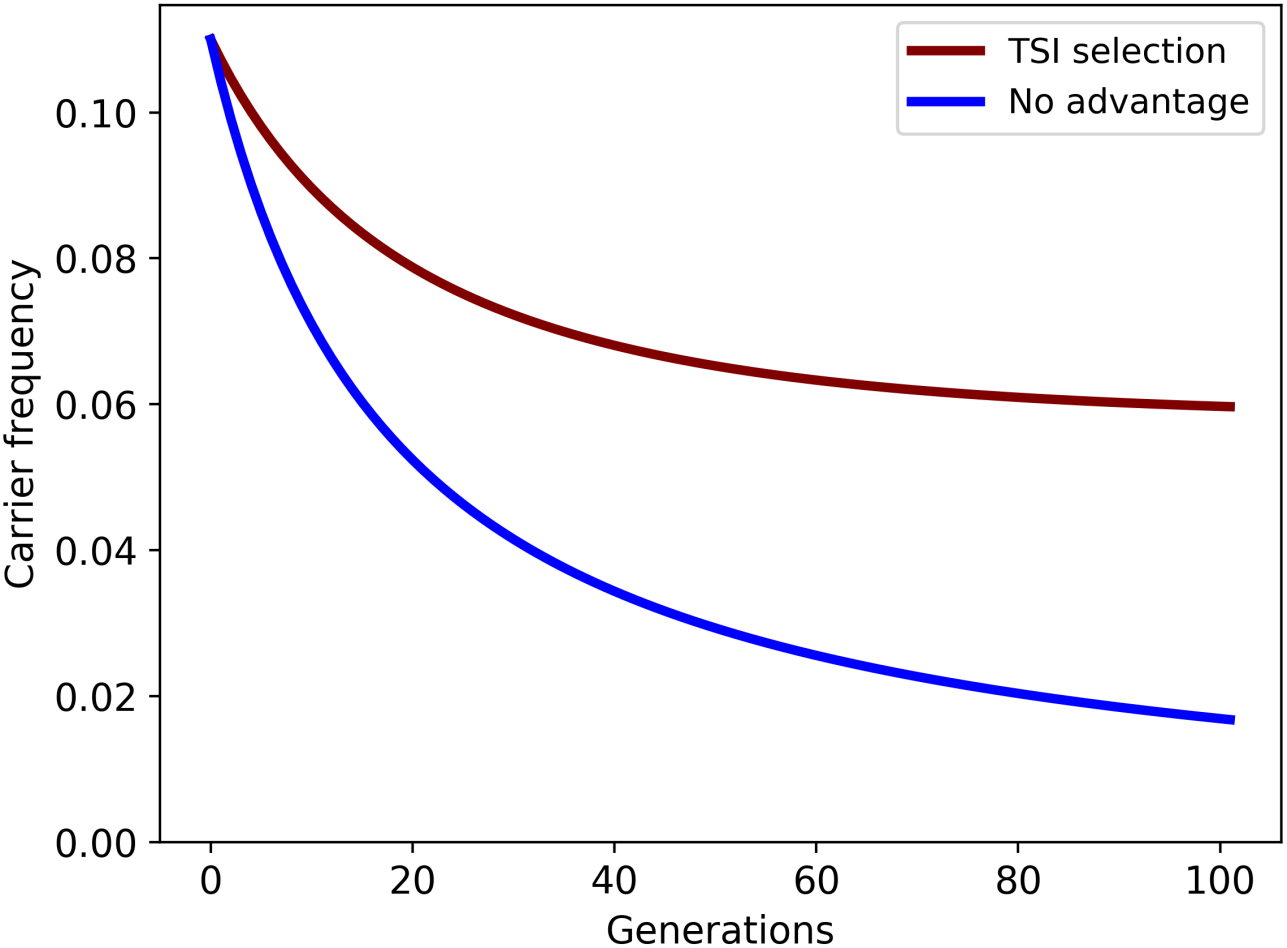
Simulation of the SSC18 carrier frequency with current selective advantage over 100 generations starting with current carrier frequency (11%). Figure shows a decrease in carrier frequency in the first generations due to the loss of homozygotes which outweighs the heterozygous advantage (~3%) perceived in the selection index (TSI). Figure shows a trade-off at approximately 6% carrier frequency at which the heterozygous advantage is compensated by the loss of homozygous offspring.

**Fig 5:**
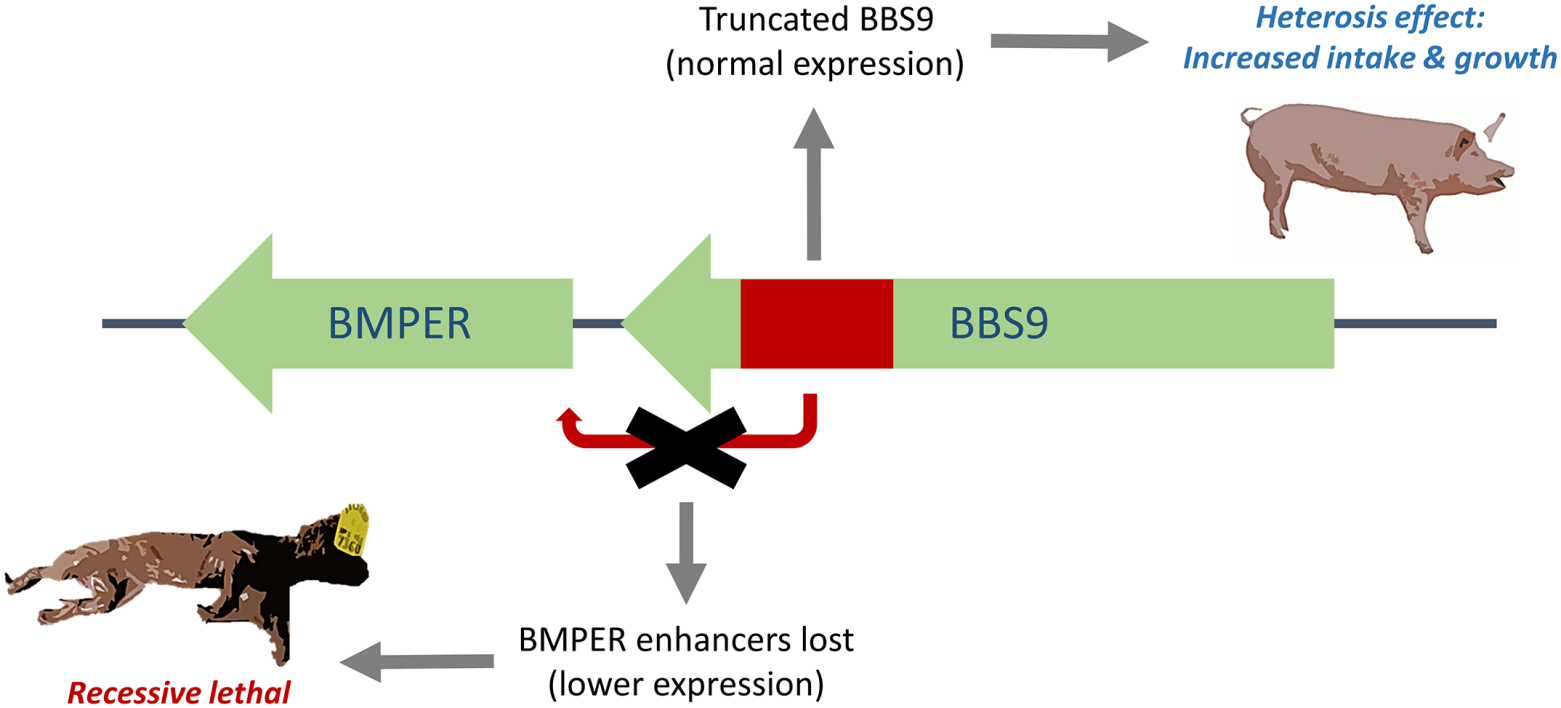
Schematic representation of the SSC18 deletion affecting *BMPER* gene expression and *BBS9* protein structure. A heterozygous loss of function of the *BBS9* gene results in increased growth rates, while reduced expression of the *BMPER* gene results in foetal mortality in homozygous del/del animals.

The carriers grow faster (TGR and LGR), have smaller loin depth (LDE), produce litters that are lighter (LBW), show higher mortality in their litters (LMO) and have a higher feed intake (DFI) when compared to the non-carriers. Selection on growth has not significantly changed in the last decade, and there is consistent increase in genetic progress for growth in this time period (S8 Fig). To further support a balancing scenario, we evaluated the difference in the total selection index (TSI) between the carrier and non-carrier group for all animals born in 2017. Animals are ranked based on this selection index to select the top animals to produce the next generation. We observe a 2.7% higher TSI (on average) for carriers compared to non-carriers (S11 Table), caused by the positive effect on growth, that outweighs the negative effect on other traits. Next, we simulated the long term effect on the SSC18 carrier frequency based on the current heterozygous advantage and frequency (Fig 4, S9 Fig). We observe a decrease in carrier frequency in the first generations due to the loss of homozygotes, which outweighs the heterozygous advantage perceived in the selection index. However, at approximately 6% carrier frequency, the heterozygous advantage compensated for the loss of homozygous offspring, reaching a trade-off at this point. Moreover, carriers show 12.4% higher breeding values for growth compared to non-carriers (S11 Table), and we show that the carrier frequency can rise up to 22% if selection would be exclusively on growth (S10 Fig). Together these results support a balancing selection scenario showing heterozygote advantage for growth rate (Fig 5), an important selection trait in the pig breeding industry.

## Discussion

Livestock populations with small effective population size can lead to the spread of recessive lethal alleles, in which the effects of drift tend to dominate over the effect on selection [25]. On the other hand, purging lethal alleles is more efficient in populations with small effective population size, which seems to contradict the relative lack of purging for the current allele under study over such long time period (almost two decades). One possible—partial—explanation is that fertility, a complex trait, is influenced by many genetic and environmental factors. As a consequence, fertility traits generally have low heritability’s [26]. One of the important determinants of fertility is prenatal mortality [27]. However, prenatal mortality caused by recessive genetic defects is often difficult to capture within the pig breeding values (smaller litters will only be generated if two carriers mate), especially if the lethal variant is segregating at relative low frequency in the population.

We report a 212kb deletion that causes death of homozygous fetuses, and which also shows a positive effect on growth rate and feed intake in heterozygous pigs (Fig 5). We show that the allele has been segregating in the population for at least 18 years, despite its detrimental lethal effects in its homozygous state. The frequency of the deleterious allele was higher a decade ago, likely caused by genetic drift, because no significant changes in selection pressure for production traits have been applied during this time period. The balancing nature of the allele is clear from the higher TSI index of carrier animals. The 212kbp deletion clearly shows a net positive effect on the chances of pigs becoming selected in the breeding program, since the TSI is used to rank selection candidates. Although lower fertility is not captured in the TSI or even breeding values related to fertility directly, it is clear that the lower number of piglets born from CxC matings will result in a decreased fitness of these crosses. As a consequence, a balance is expected to arise between positive and negative selection, and indeed, the heterozygous advantage captured in the selection index compensates for the loss of homozygotes at approximately 6% carrier frequency. This frequency is somewhat lower than the current and past observed frequency. One partial explanation for this discrepancy is that we overestimate the number of CxC matings in our simulations (assuming random matings), because in practice matings between related individuals are avoided. Furthermore, during the 20^th^ century, selection was mainly applied on growth and carcass traits [28], indicating that the heterozygote advantage for carriers was likely stronger in the past. Interestingly, the balance between positive and negative selection would explain a rapid increase in the population under strong selection for growth traits, as has been the case for pig breeding lines at least since the introduction of modern breeding techniques.

This increase in growth rate (with the most significant effect in the test period from 25–120 Kg) and feed intake, likely results from a heterozygous loss-of-function of the *BBS9* gene. The BBS9 protein is part of the BBSome complex, and is required for ciliogenesis [29]. BBS9 is the central organizational component of the BBSome, having direct interactions with BBS1, 2, 5 and 8 [30]. Loss-of-function mutations in human *BBS9* and other members of the BBSome cause Bardet-Biedl syndrome, associated with a series of clinical features including obesity, renal anomalies, and retinopathy, with the obese phenotype as one of the key features of Bardet-Biedl syndrome patients. Studies have hypothesized that cilia defects are likely to affect feeding and satiety, causing an increased appetite and lack of satiation [29]. In addition, heterozygous carriers of a mutant BBS allele in humans show increased levels of obesity, without showing any of the other Bardet-Biedl syndrome features [31], analogous to the observed phenotype in carriers of the *BBS9* deletion. Mouse null-mutants in genes that form the BBSome complex have been associated with similar phenotypic features including obesity, lower birth weights, and partial embryonic lethality [32, 33], again supporting the *BBS9* role in increased growth rate, and the lower birth weight. We cannot completely exclude, however, that other genomic factors, in high LD with the 212kb deletion, contribute to the observed phenotype as well.

The question remains which gene or regulatory element is causal for the early death of homozygous individuals. We expect that the deletion (in homozygous state) leads to a complete loss-of-function of the *BBS9* gene, and decreased expression of the *BMPER* gene by affecting *BMPER* enhancer elements. Enhancers are important drivers of transcription and loss of enhancer elements can lead to decreased expression of the associated gene. Naturally occurring knock-outs of the *BBS9* gene does not result in fetal lethality in human [18] and is therefore not likely to be causal for the lethal phenotype. Instead, the downstream *BMPER* gene is a much stronger candidate since *BMPER* null mutants result in prenatal lethality with skeletal malformations in both mice and human [34, 35], marking the *BMPER* gene as the likely candidate underlying this phenomenon. We hypothesize that the deletion affects *BMPER* enhancer elements, resulting in insufficient expression of the *BMPER* gene in homozygous state. The lower expression of the BMPER gene is supported by allele specific expression for the non-deletion haplotype in carriers, not observed in individuals only carrying wild-type haplotypes. However, other BBS proteins, part of the BBSome, do cause (partial) embryonic lethality (i.e. *BBS4* [33] and *BBS7* [36]), we can therefore not exclude the possibility that a complete loss of a functional BBS9 protein contributes to the early lethality as well. Together these results support that the deletion affects *BMPER* regulatory elements resulting in allelic imbalance for the *BMPER* gene in carrier animals. Therefore, severe downregulation of the *BMPER* gene is expected for del/del animals causing fetal death.

This work describes a striking example of balancing selection in pigs, maintaining a recessive lethal allele that shows pleiotropic effects on fertility and growth traits at moderate frequency in the population. Other examples in pigs include the Porcine Stress and Pale Soft Exudative Meat Syndrome, caused by a homozygous missense mutation in the *RYR1* gene, while heterozygotes show increased muscle mass [37]. A second example is a LINE insertion in the *SPEF2* gene causing increased litter size in sows but decreased fertility in boars [16]. Moreover, several instances of balancing selection have been described in domestic cattle breeds among which a 660kb deletion causing embryonic lethality in homozygotes, while having increased milk yield in heterozygotes [5, 15]. Identifying balancing selection on lethal alleles can be challenging, as the only consequence observed is a (somewhat) lower fertility in the parental animals, lacking affected (liveborn) individuals. We expect that this type of balancing selection might be more prevalent within pig populations than previously thought, especially for the somewhat higher frequency lethal alleles, which are less likely to be purely the result of drift effects. Moreover, the relatively subtle effects found in this study could only be made apparent because phenotypic data derived from a very large number of pigs was available.

### Conclusion

In this study we report a 212 kb deletion with antagonistic effects on fertility and growth. We show that homozygotes for the deletion die around mid- to late-gestation, becoming mummified. Compared to other lethal alleles identified in this population, the deletion seems to be maintained at moderate frequency (10.8%) in the population. This moderate carrier frequency is likely not a result of random drift effects, as heterozygotes for the deletion-haplotype show, despite a lower birth weight, increased growth rate, and feed intake, important traits in the breeding goal. The balancing scenario observed, most likely, is a consequence of pleiotropic effects of the deletion on two different genes affecting fertility (*BMPER*) and growth (*BBS9*). The large amount of genotype data accumulating in modern breeding schemes applying genomic selection in combination with the large amount of phenotypic data deliver a powerful tool to monitor and control deleterious alleles much more efficiently.

## Material and methods

### Ethics statement

Samples collected for DNA extraction were only used for routine diagnostic purpose of the breeding programs, and not specifically for the purpose of this project. Therefore, approval of an ethics committee was not mandatory. Sample collection and data recording were conducted strictly according to the Dutch law on animal protection and welfare (Gezondheids- en welzijnswet voor dieren).

### Animals, genotypes and pre-processing

The dataset consists of 23,722 purebred Large White animals. The animals were genotyped on the Illumina GeneSeek custom 50K SNP chip (Lincoln, NE, USA). Animals with a frequency of missing genotypes > 0.20 were removed. We discard markers that did not meet following filtering criteria: A minimum call rate of 0.85, a minor allele frequency > 0.01, and a Hardy-Weinberg proportions exact test p-value below P < 10^−6^. Moreover, markers with unknown location on the Sscrofa11.1 genome build [38] were discarded, leaving 42,288 markers after filtering. All steps were performed in Plink v1.90b3.30 [39].

### Haplotype phasing

We performed haplotype phasing and imputation of missing sites in Beagle4.1 with parameter for effective population size set to 195, other settings were default [40]. Reference and test phased VCF files were merged using bcftools 1.3–27-gf31e888 [41].

### Identification of missing homozygote haplotypes

We tested the SS18 haplotype for the expected number of homozygotes using both parents haplotype information (sire, and dam) with the formula described in Fritz et al., 2013 [42]. An exact binomial test was applied to test the number of observed homozygotes with the number of expected homozygotes. The haplotype was considered significantly depleted if P < 5 × 10^−3^. The difference in Mendelian ratios for CxC compared to CxNC matings was tested using a Chi-Square test.

### Pseudo genotyping for SSC18 deletion

To genotype animals directly for the SSC18 deletion, we first calculated LRR normalized signal intensities using PennCNV analysis software [43]. We built a classifier with 5 features: the LRR signal intensities for the four overlapping markers within the deletion (WU_10.2_18_43630319, WU_10.2_18_43773633, WU_10.2_18_43778188, WU_10.2_18_43803484), and the average LRR signal intensity over these four markers. Next, we applied logistic regression to distinguish carrier from non-carrier animals using the sci-kit learn Python library [44] (S6 Fig).

### Phenotypic effects associated with lethal haplotypes

We examined the SSC18 haplotype for records on TNB, NSB, and MUM listed for all C x C, and C x NC matings identified in the phenotypic records, the order of C x NC matings does not reflect the sex of the parent animal and is both carrier boar and carrier sow combined. We used a Welch’s t-test to assess whether the phenotypes from the C x C matings differ significantly from C x NC matings. A p-value < 0.05 was considered significant.

### WGS analysis and candidate variant identification

The dataset consists of 73 whole genome sequenced Large White individuals with a total volume of 1.77 Tbp (tera base pairs) from 15.539 billion paired-end reads, ranging from 100–150 bp in length (S5 Table). The data was sequenced on Illumina Hiseq 2000. We used sickle software for quality trimming of raw reads. Next we aligned the sequences to the Sscrofa11.1 genome build [38] using BWA-MEM version 0.7.15 [45] with an average mappability of 96.11% and a sample coverage ranging from 6.6–22.7X (10X average). Samtools dedup function was used to remove PCR duplicates [41]. GATK IndelRealigner was used to perform local realignments around indels [46]. Variant calling was performed with Freebayes v1.1.0 with following settings:—min-base-quality 10—min-alternate-fraction 0.2—haplotype-length 0—min-alternate-count 2 [47]. Variants with phred quality score < 20, and within 3 bp of an indel were discarded [41]. Variants were annotated using the Ensembl variant effect predictor (VEP, release 90) [17]. The impact of missense variants was predicted using SIFT [48]. The sequenced population was phased using Beagle4.1 [40].

### Structural variation analysis

Analysis on structural variation (SV) was performed using Lumpy with default settings [49], taking the aligned BAM files as input. Coverage information was calculated for predicted SV events using samtools depth [41], and added to the VCF format tag using PyVCF. Alignments and SV events were visualized using the JBrowse genome viewer version 1.12.1 [50].

### RNA-seq analysis and allele specific expression

We analyzed RNA-seq data on eight different tissues in one SSC18 carrier animal (sample: PigWUR166). In addition, we analyzed two other pigs from Duroc, and Pietrain genetic background on five different tissues. RNA-seq reads were aligned to the Sscrofa11.1 genome build using STAR 2.5.3a [51], generation of transcripts and gene expression levels were achieved with Cufflinks v2.2.1 [52]. We applied the following steps to examine allele specific expression: First, samtools [41] was used to extract uniquely mapped reads from the BAM alignment files. Next, WASP [53] was used to reduce the mapping (reference sequence) bias. Then, GATKASEreadcounter [46] was used to obtain read counts for reference and alternative alleles at each SNP position. Lastly, a two-sided binomial test with p = 0.5 (assuming no bias) and Benjamini-Hochberg false discovery rate (FDR) correction were performed in R v.3.4 at each variant position using the Stats package. The variants with FDR adjusted p-value < 0.05 were considered as allele specific expression variants. Visual examination of the alignments and transcripts was performed in JBrowse [50].

### RNA-isolation and RT-qPCR

RNA was extracted from frozen whole blood using the Nucleospin RNA blood kit from Machery Nagel. cDNA was synthesized using Superscript II Reverse Transcriptase (Invitrogen) with RNA input ~100ng. RT-qPCR was started with: 3.75ul cDNA (1:1), 1.25ul primer forward (2uM), 1.25ul primer reverse (2uM), and 6.25ul MESA blue mix (Eurogentec). RT-qPCR was then performed with a QuantStudio 5 system using the comparative Ct (delta delta Ct) method with GAPDH as housekeeping gene for normalization. Reaction was performed as follows:

1. 50°C 2 min, 1 cycle
2. 95°C 10 min, 1 cycle
3. 95°C 15 sec, 1 cycle
4. 60°C 1 min, 40 cycles
5. 95°C 15 sec, 1 cycle
6. 60°C 1 min, 1 cycle
7. 95°C 15 sec, 1 cycle

Data was analysed with the Quantstudio Design & Analysis Software v.1.4.3. All primers and results are listed in S12 and S13 Tables.

### ChipSeq alignment

We downloaded three H3K27Ac, and three H3K4me3 libraries (ArrayExpress accession number: E-MTAB-2633) from liver tissue from three male pig samples described by Villar et al. 2015 [21]. Data was aligned using BWA-mem [45] and visualized in JBrowse [50].

### Frequency over time

We analyzed the frequency of the SSC18 haplotype per half-year starting from 01-jul-2006. We assessed the frequency based on total population (live animals) on each time point by looking at the proportion of carrier and non-carrier animals in the population. The number of animals per time point are provided in S8 Table. We used a One-Way Repeated Measures ANOVA to test whether the frequency differs over time.

### Breeding values and association analysis

In this study, we evaluated 16 traits used in the Large White breeding program. Deregressed estimated breeding values (DEBV) were used as a response variable for each trait under study. The estimated breeding value (EBV) was separately deregressed for each trait using the methodology described by Garrick et al [54]. The EBV of each animal was obtained from the routine genetic evaluation by Topigs Norsvin using an animal model. The reliabilities per animal for the purpose of deregression were extracted from the genetic evaluation based on the methodology of Tier & Meyer [55]. The heritabilities used for the deregression were also extracted from the routine genetic evaluation. Parent average effects were also removed as part of the deregression process to obtain more accurate estimates of the genetic merit of each individual. Finally, weighting factors based on the estimated reliability of the DEBV were also estimated according to Garrick et al [54] using a value of 0.5 for the scalar c. To ensure the quality of the DEBV, only animals with a w higher than not equal to zero and a reliability of the DEBV greater than 0.20 were used in the association analyses. The reliability of the DEBV was obtained according to Garrick et al [54].

Association analyses were performed using the software ASREML [56] applying the following model:

$$DEBV_{ij}\omega \;=\;\mu +R_{i}+a_{j}+e_{ij,}$$

where DEBV_*ij*_ is the observed DEBV for the animal *j*, w is weighting factor for the residual, *μ* is the overall DEBV mean of the population, *R*_*i*_ is the carrier status of the lethal allele *i*, *a*_*j*_ is the additive genetic effect estimated using a pedigree-based relationship matrix, and *e*_*ij*_ the residual error.

### Simulating genetic drift

We simulated changes in allele frequency across multiple populations under the model of Wright [57]. Each allele is associated with a fitness, and we set the fitness to zero for homozygotes (for lethal recessive allele) and fitness to 1 (no negative fitness effect) for carriers and non-carriers. We assume constant population size through time, and matings are simulated randomly at each generation. Changes in allele frequencies are plotted using the R package driftR (https://github.com/cjbattey/driftR).

### Balancing selection

Within each generation the top 5% of boars, and top 25% of gilts (based on the TSI selection index value) are used to produce the next generation. We first calculated the average TSI, and estimated breeding values for six important traits in the breeding line (S11 Table). Next, we used the ratio of carrier TSI over non-carrier TSI to estimate the selective advantage in the breeding program. Next, we simulated the long-term allele frequency change (assuming random matings) based on the selective advantage, and the loss of homozygous animals using the Hardy-Weinberg principle. Similar analysis was performed using the selective advantage on growth exclusively.

## Supporting information

S1 Fig Pictures and X-rays from mummified piglets. A: Picture of a homozygous del/del mummified piglet (7360). B: X-ray of homozygous del/del mummified piglet (7360). C: X-ray of mummified piglet 7659 (status unknown). D: X-ray of homozygous del/del mummified piglet (8609).
(PDF)

S2 Fig Screen capture of the deletion boundary in the JBrowse genome browser. (PDF)

S3 Fig LRR signal intensities within the haplotype region for two "fresh born" homozygotes of the 212kb deletion. (PDF)

S4 Fig Expression fold change of the *BBS9* gene (RT-qPCR) in 8 carriers, and 10 non-carriers. (PDF)

S5 Fig JBrowse screen capture showing aligned liver ChipSeq data (H3K27Ac, H3K4Me3) on Sscrofa11.1. (PDF)

S6 Fig Logistic regression to distinguish carrier from non-carrier animals (farm 2 litters). (PDF)

S7 Fig Genetic drift simulation for SSC18 lethal recessive with allele frequency 5.4% (10.8% carrier frequency). (PDF)

S8 Fig Genetic progress for growth (daily gain) in the Large White breed. (PDF)

S9 Fig Simulation of the SSC18 carrier frequency with current selective advantage over 200 generations starting with lower carrier frequency (2%). (PDF)

S10 Fig Simulation of the SSC18 carrier frequency if selection would be exclusively applied on growth. (PDF)

S1 Table Results from two CxC (CC1: 11-may-2017, CC3: 19-may-2017) matings on farm 1. (PDF)

S2 Table Results from two CxC (CC2: 18-may-2017, CC4: 01-jul-2017) matings on farm 2. (PDF)

S3 Table Markers and genomic positions. (XLSX)

S4 Table Haplotypes from the four tracked carrier-by-carrier matings, including parent animals. (XLSX)

S5 Table WGS sequenced individuals in the Large White breed. (PDF)

S6 Table Gene expression measured in fragments per kilobase per million (FPKM) for BBS9 and BMPER gene in one SSC18 deletion carrier animal (LW) and two non-carrier pigs (DU/PI). (PDF)

S7 Table Allele specific expression test of the BMPER gene for three non-carriers pigs. Heterozygous coding variants of the BMPER canonical transcript (XM_013990842.2) are used to test for allelic imbalance.
(PDF)

S8 Table Deletion carrier frequency and the number of genotyped animals per time point from 2006–2018. (PDF)

S9 Table Dumeco-W carriers for SSC18 deletion including birth dates. (PDF)

S10 Table Association analysis using deregressed breeding values (DEBV) for 16 traits in the Large White breed. (PDF)

S11 Table Estimated breeding values (EBV) for seven traits including the overall selection index (TSI). (PDF)

S12 Table Primer information for the genes used for RT-qPCR. (PDF)

S13 Table RT-qPCR results for *BBS9* expression in 8 carriers and 10 non-carriers. (PDF)
