# Supplementary material for: Balancing selection on a recessive lethal deletion with pleiotropic effects on two neighboring genes in the porcine genome
Source: PLoS Genet. 2018 Sep 19;14(9):e1007661. doi: 10.1371/journal.pgen.1007661 (PMC6166978; doi:10.1371/journal.pgen.1007661)
Supplement: S1 Fig — A: Picture of a homozygous del/del mummified piglet (7360). B: X-ray of homozygous del/del mummified piglet (7360). C: X-ray of mummified piglet 7659 (status unknown). D: X-ray of homozygous del/del mummified piglet (8609). (PDF) [file pgen.1007661.s001.pdf]

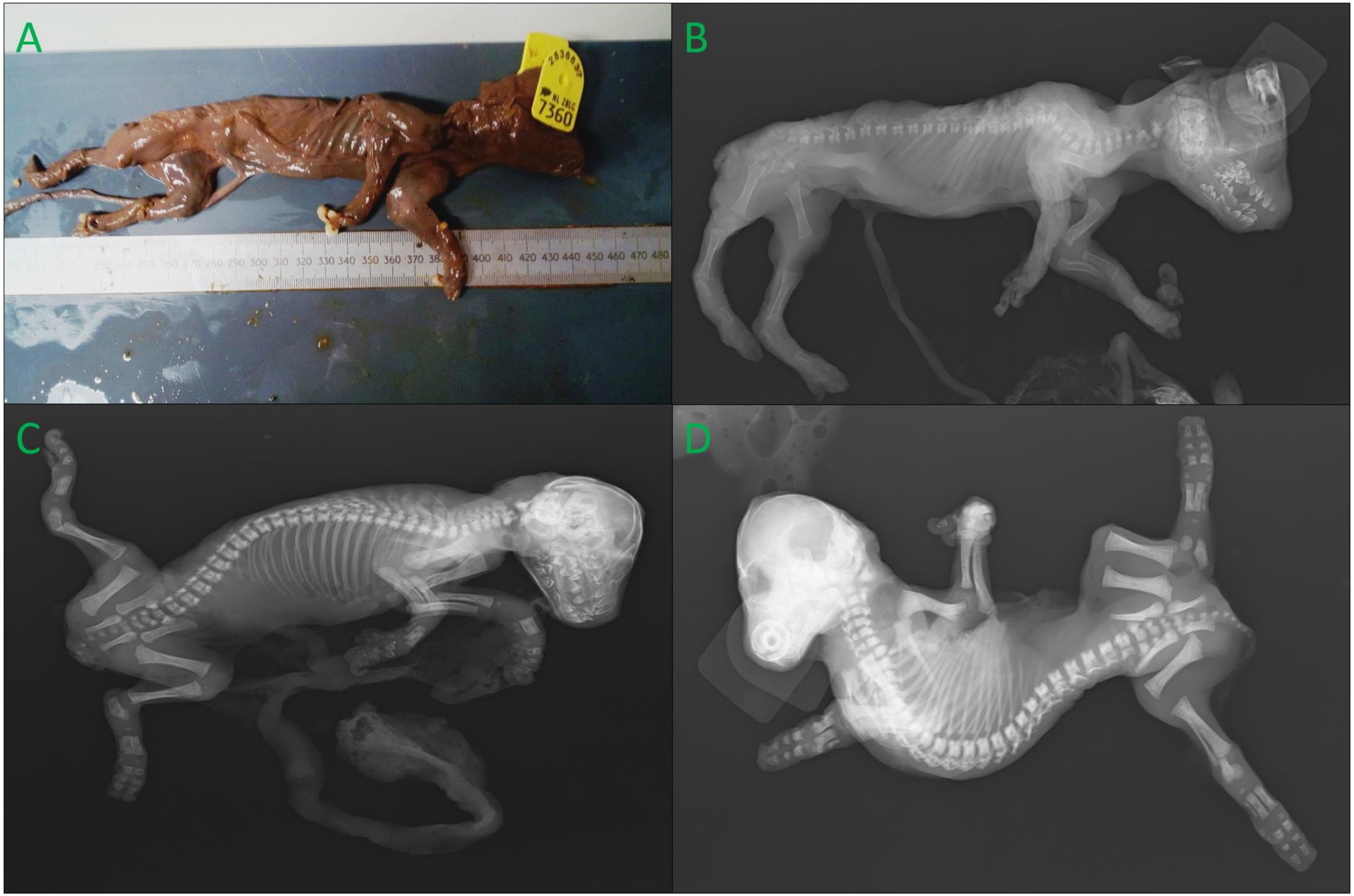

Figure S1: Pictures and X-rays from mummified piglets. A: Picture of a homozygous del/del mummified piglet (7360). B: X-ray of homozygous del/del mummified piglet (7360). C: X-ray of mummified piglet 7659 (status unknown). D: X-ray of homozygous del/del mummified piglet (8609).
