# Supplementary material for: Balancing selection on a recessive lethal deletion with pleiotropic effects on two neighboring genes in the porcine genome
Source: PLoS Genet. 2018 Sep 19;14(9):e1007661. doi: 10.1371/journal.pgen.1007661 (PMC6166978; doi:10.1371/journal.pgen.1007661)
Supplement: S2 Fig — (PDF) [file pgen.1007661.s002.pdf]

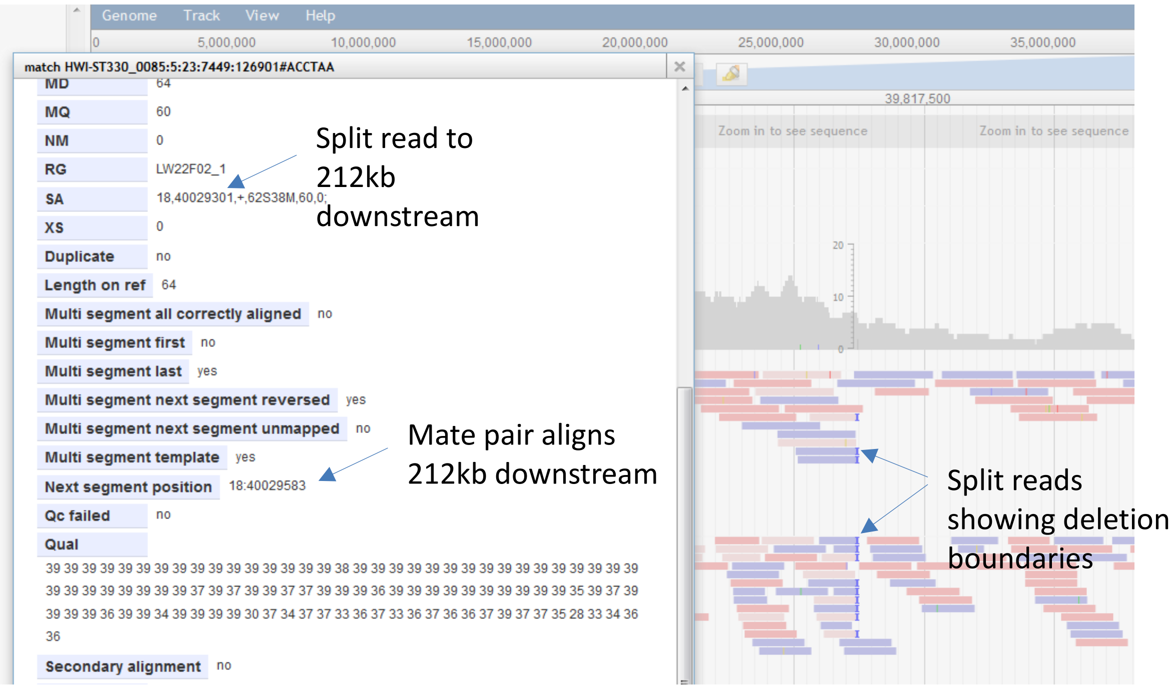

Figure S2: Screen capture of the deletion boundary in the JBrowse genome browser. Figure shows split-, and discordant read mappings on the boundaries of the 212kb deletion.
