# Supplementary material for: Balancing selection on a recessive lethal deletion with pleiotropic effects on two neighboring genes in the porcine genome
Source: PLoS Genet. 2018 Sep 19;14(9):e1007661. doi: 10.1371/journal.pgen.1007661 (PMC6166978; doi:10.1371/journal.pgen.1007661)
Supplement: S3 Fig — (PDF) [file pgen.1007661.s003.pdf]

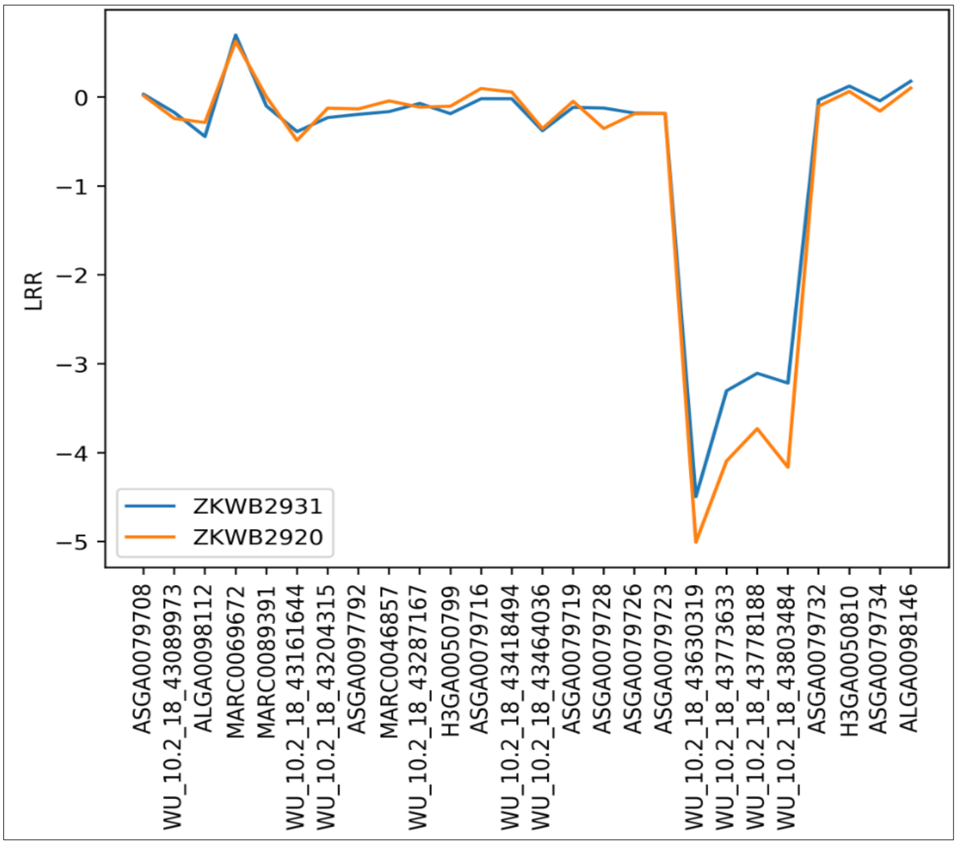

Figure S3: LRR signal intensities within the haplotype region for two "fresh born" homozygotes of the 212kb deletion. ZKWB2931 was a stillborn piglet, while ZKWB2920 was born alive, but it was very weak and died shortly after birth.
