# Supplementary material for: Balancing selection on a recessive lethal deletion with pleiotropic effects on two neighboring genes in the porcine genome
Source: PLoS Genet. 2018 Sep 19;14(9):e1007661. doi: 10.1371/journal.pgen.1007661 (PMC6166978; doi:10.1371/journal.pgen.1007661)
Supplement: S4 Fig — (PDF) [file pgen.1007661.s004.pdf]

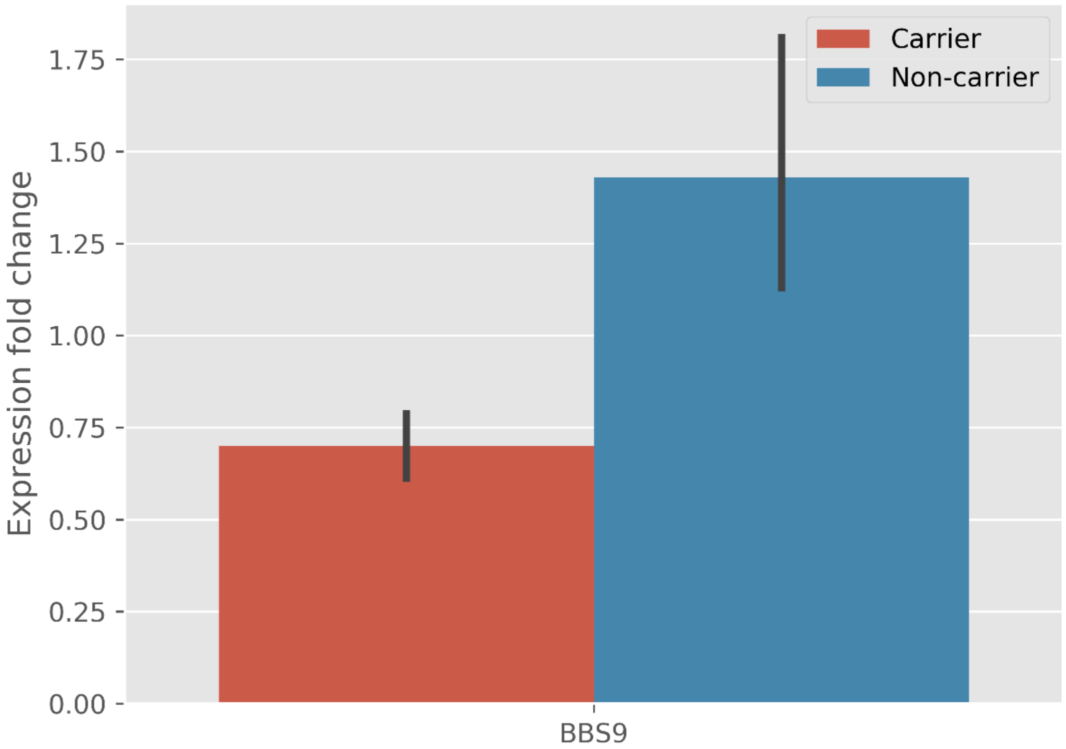

Figure S4: Expression fold change of the *BBS9* gene (RT-qPCR) in 8 carriers, and 10 non-carriers. Figure shows a two-fold lower expression of the wild-type *BBS9* gene in carriers compared to non-carriers.
