# Supplementary material for: Balancing selection on a recessive lethal deletion with pleiotropic effects on two neighboring genes in the porcine genome
Source: PLoS Genet. 2018 Sep 19;14(9):e1007661. doi: 10.1371/journal.pgen.1007661 (PMC6166978; doi:10.1371/journal.pgen.1007661)
Supplement: S5 Fig — (PDF) [file pgen.1007661.s005.pdf]

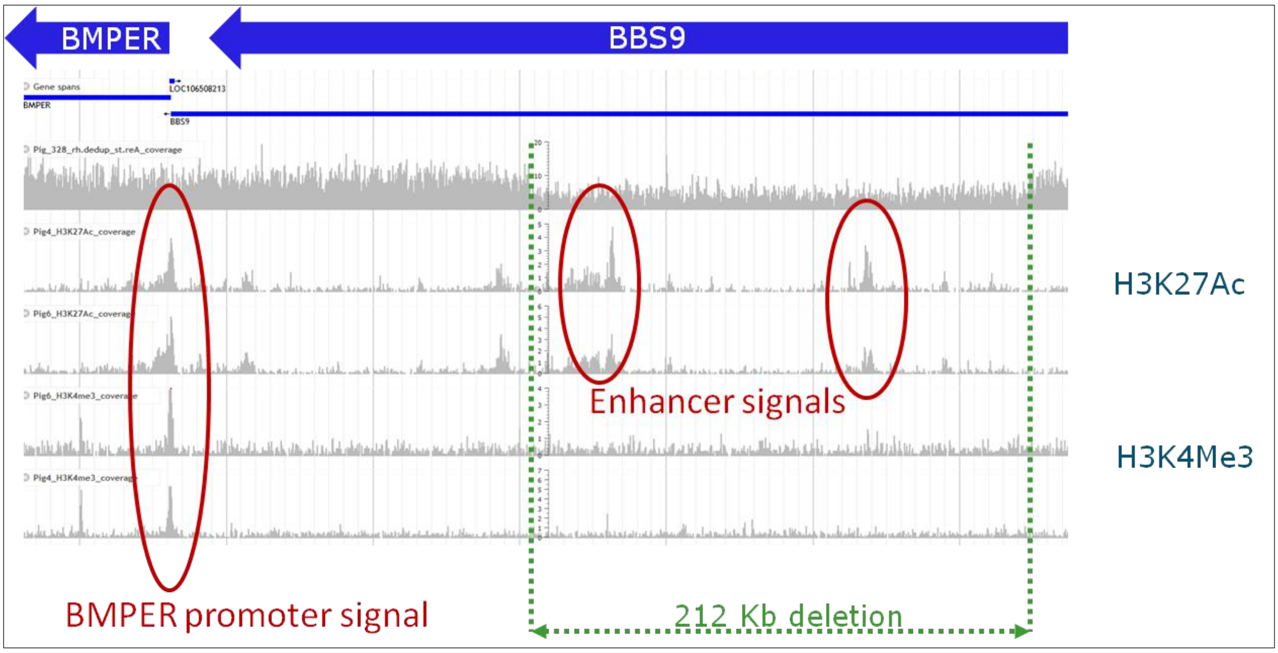

Figure S5: JBrowse screen capture showing aligned liver ChipSeq data (H3K27Ac, H3K4Me3) on Sscrofa11.1 (Villar et al. 2015). Figure shows two enhancer signals in the deletion region and the BMPER promoter signal downstream of the deletion.

References

Villar D, Berthelot C, Aldridge S, Rayner TF, Lukk M, Pignatelli M, Park TJ, Deaville R, Erichsen JT, Jasinska AJ et al. 2015. Enhancer evolution across 20 mammalian species. *Cell* **160**: 554-566.
