# Supplementary material for: Balancing selection on a recessive lethal deletion with pleiotropic effects on two neighboring genes in the porcine genome
Source: PLoS Genet. 2018 Sep 19;14(9):e1007661. doi: 10.1371/journal.pgen.1007661 (PMC6166978; doi:10.1371/journal.pgen.1007661)
Supplement: S6 Fig — (PDF) [file pgen.1007661.s006.pdf]

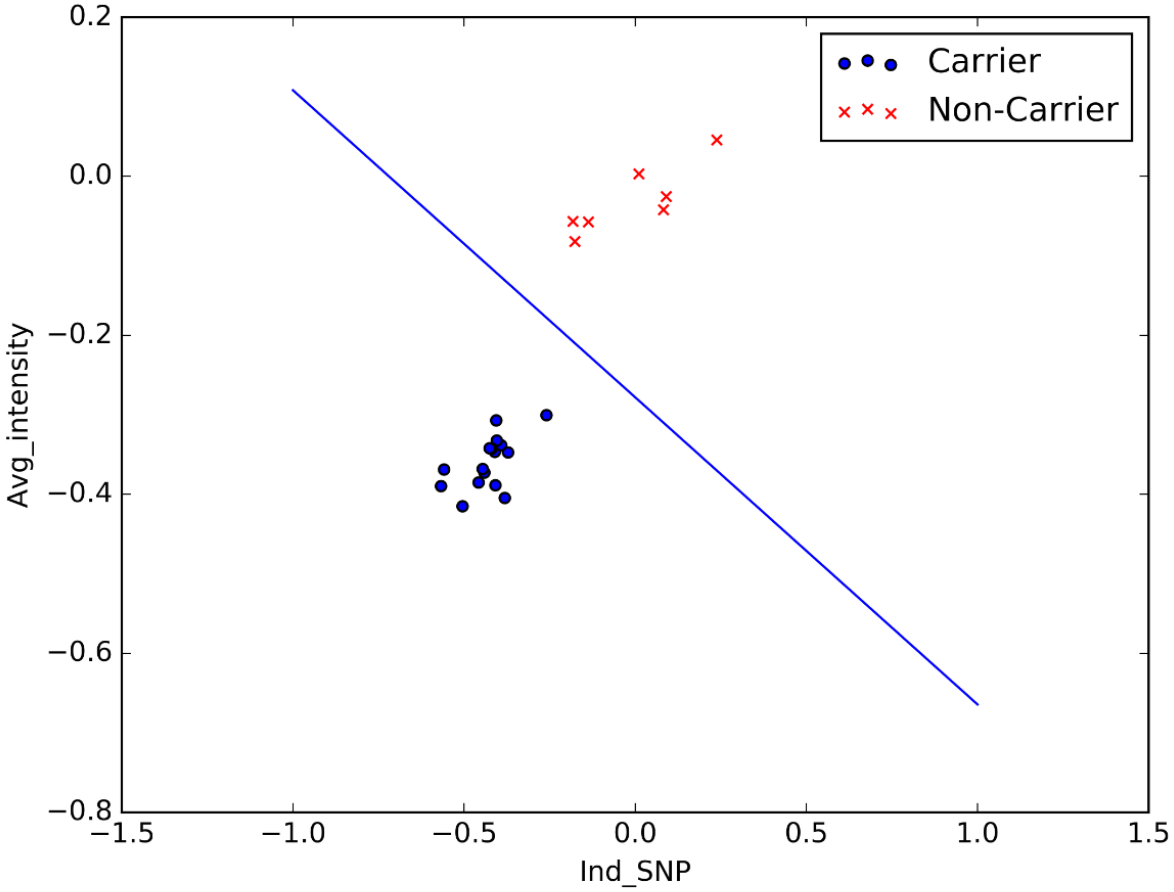

Figure S6: Logistic regression to distinguish carrier from non-carrier animals (farm 2 litters). The classifier uses the LRR signal intensities for the four overlapping markers within the deletion (WU_10.2_18_43630319, WU_10.2_18_43773633, WU_10.2_18_43778188, WU_10.2_18_43803484), and the average LRR signal intensity over these four markers. Figure shows the two most useful features to classify the two groups.
