# Supplementary material for: Balancing selection on a recessive lethal deletion with pleiotropic effects on two neighboring genes in the porcine genome
Source: PLoS Genet. 2018 Sep 19;14(9):e1007661. doi: 10.1371/journal.pgen.1007661 (PMC6166978; doi:10.1371/journal.pgen.1007661)
Supplement: S7 Fig — (PDF) [file pgen.1007661.s007.pdf]

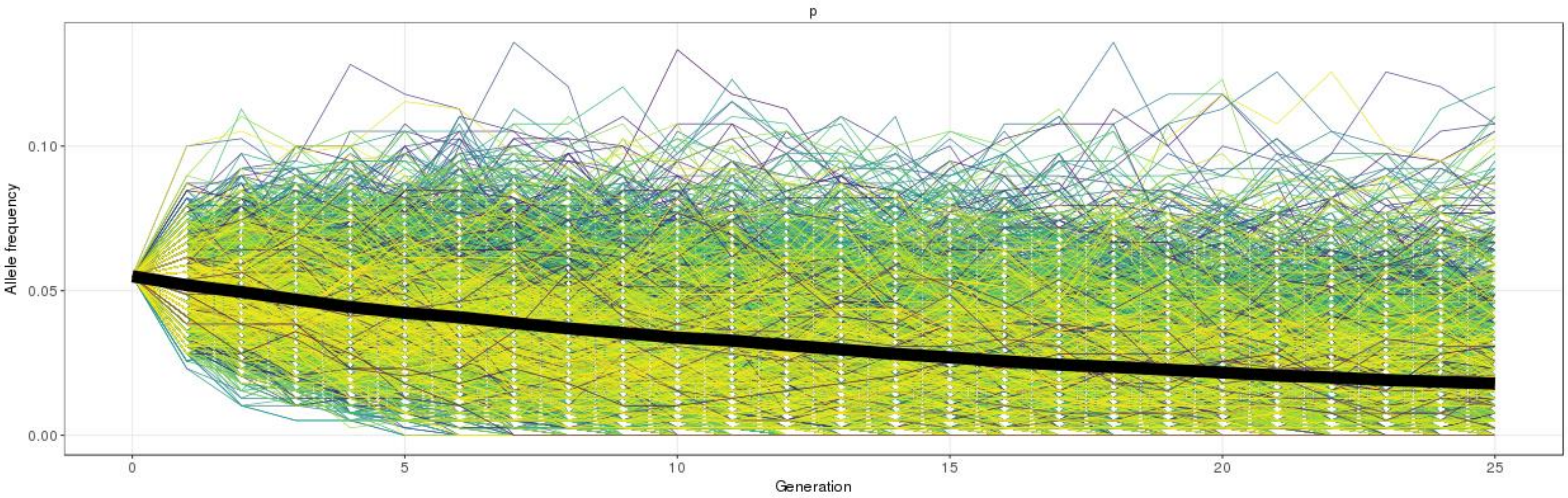

Figure S7: Genetic drift simulation for SSC18 lethal recessive with allele frequency 5.4% (10.8% carrier frequency). Plot shows frequency after 25 generations for 1000 simulations. The allele is lost in approximately 40% of the simulations. In about 11% of the simulations the frequency after 25 generations is equal or greater than the start frequency.
