# Supplementary material for: Balancing selection on a recessive lethal deletion with pleiotropic effects on two neighboring genes in the porcine genome
Source: PLoS Genet. 2018 Sep 19;14(9):e1007661. doi: 10.1371/journal.pgen.1007661 (PMC6166978; doi:10.1371/journal.pgen.1007661)
Supplement: S8 Fig — (PDF) [file pgen.1007661.s008.pdf]

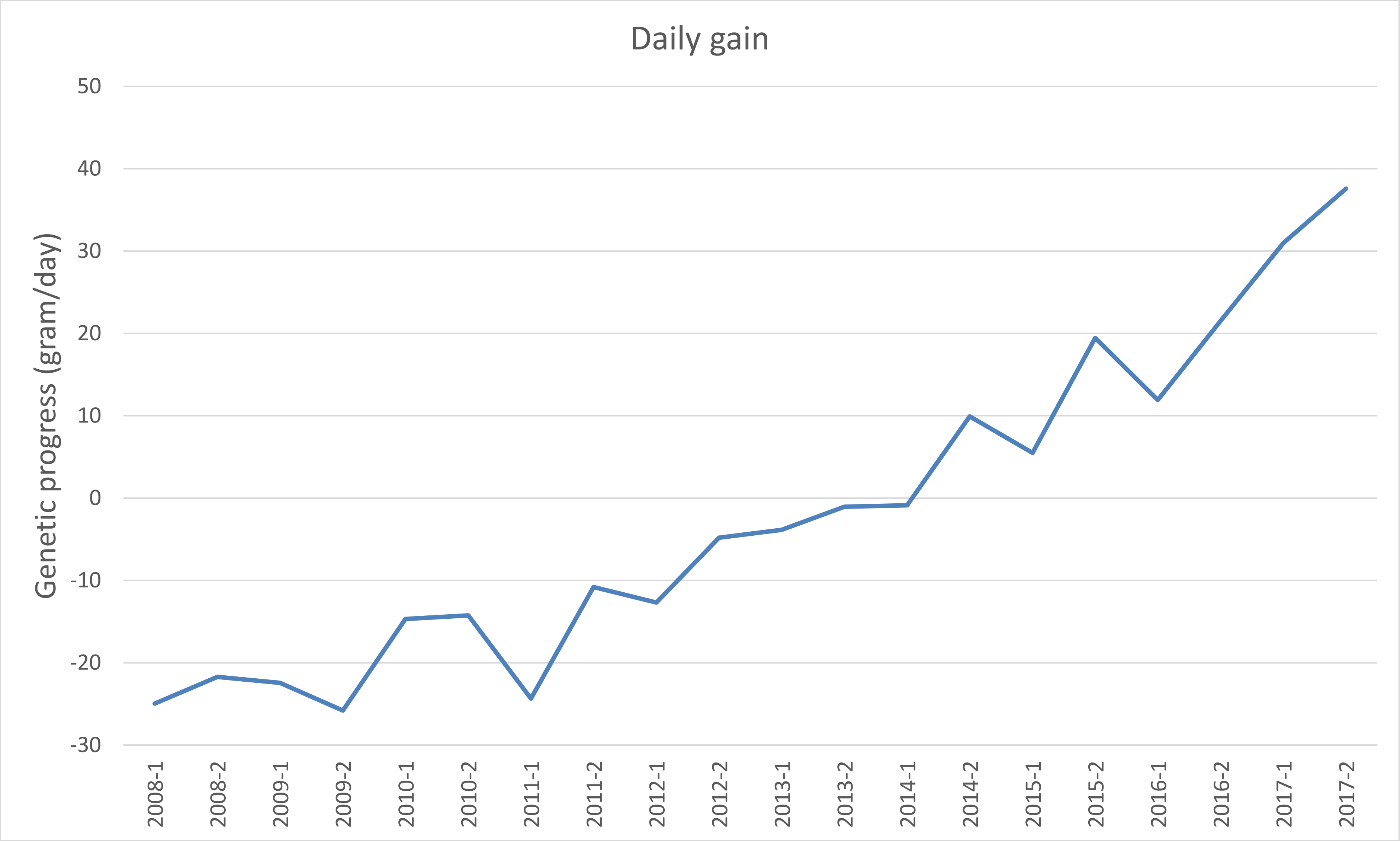

Figure S8: Genetic progress for growth (daily gain) in the Large White breed. Figure shows consistent increase in genetic progress for daily gain in the time period 2008-2017.
