# Supplementary material for: Balancing selection on a recessive lethal deletion with pleiotropic effects on two neighboring genes in the porcine genome
Source: PLoS Genet. 2018 Sep 19;14(9):e1007661. doi: 10.1371/journal.pgen.1007661 (PMC6166978; doi:10.1371/journal.pgen.1007661)
Supplement: S9 Fig — (PDF) [file pgen.1007661.s009.pdf]

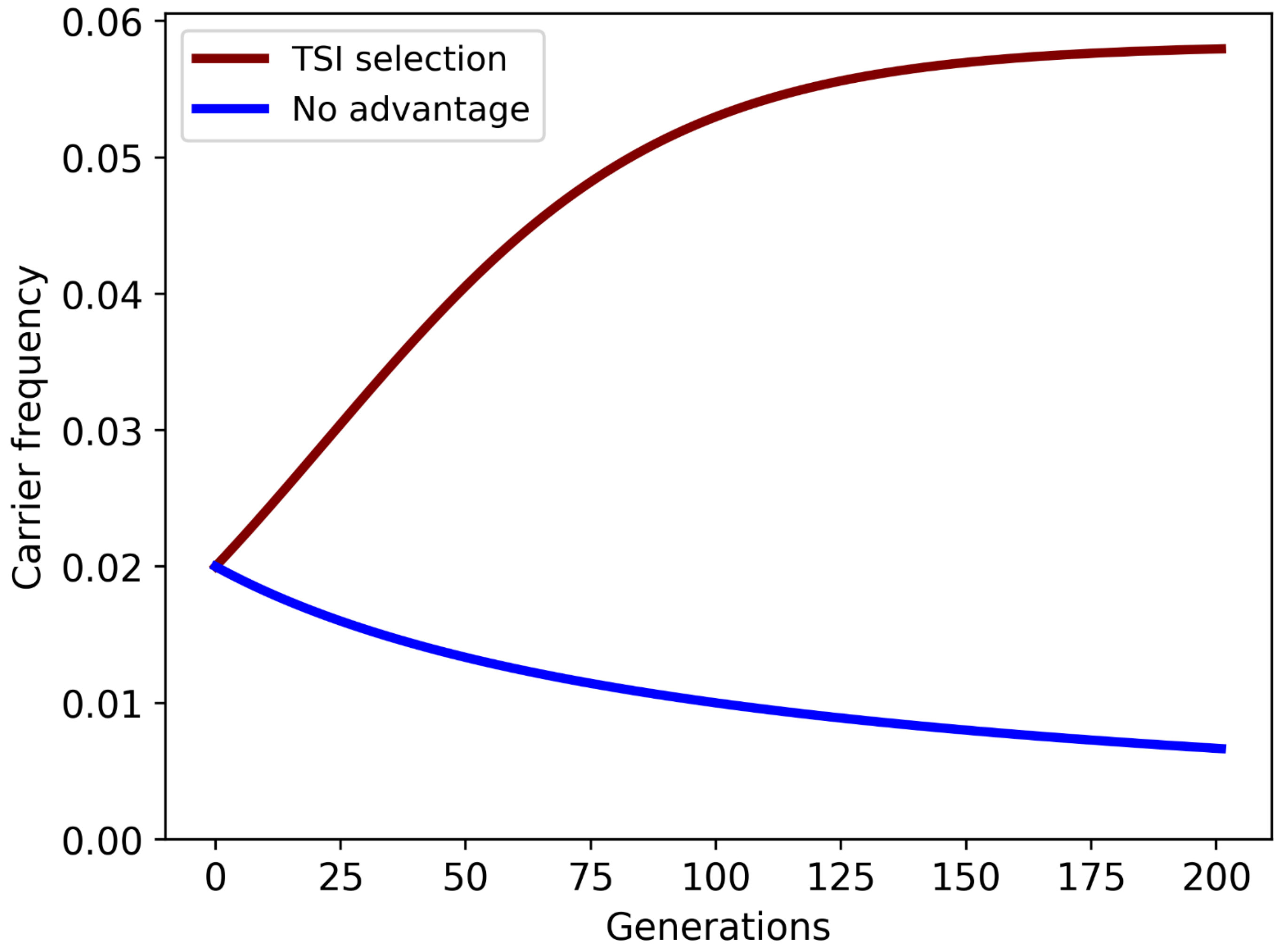

Figure S9: Simulation of the SSC18 carrier frequency with current selective advantage over 200 generations starting with lower carrier frequency (2%). Figure shows increasing carrier frequency due to heterozygous advantage (~3%) perceived in the selection index (TSI). Figure shows a trade-off at approximately 6% carrier frequency at which the heterozygous advantage is compensated by the loss of homozygous offspring.
