# Supplementary material for: Balancing selection on a recessive lethal deletion with pleiotropic effects on two neighboring genes in the porcine genome
Source: PLoS Genet. 2018 Sep 19;14(9):e1007661. doi: 10.1371/journal.pgen.1007661 (PMC6166978; doi:10.1371/journal.pgen.1007661)
Supplement: S10 Fig — (PDF) [file pgen.1007661.s010.pdf]

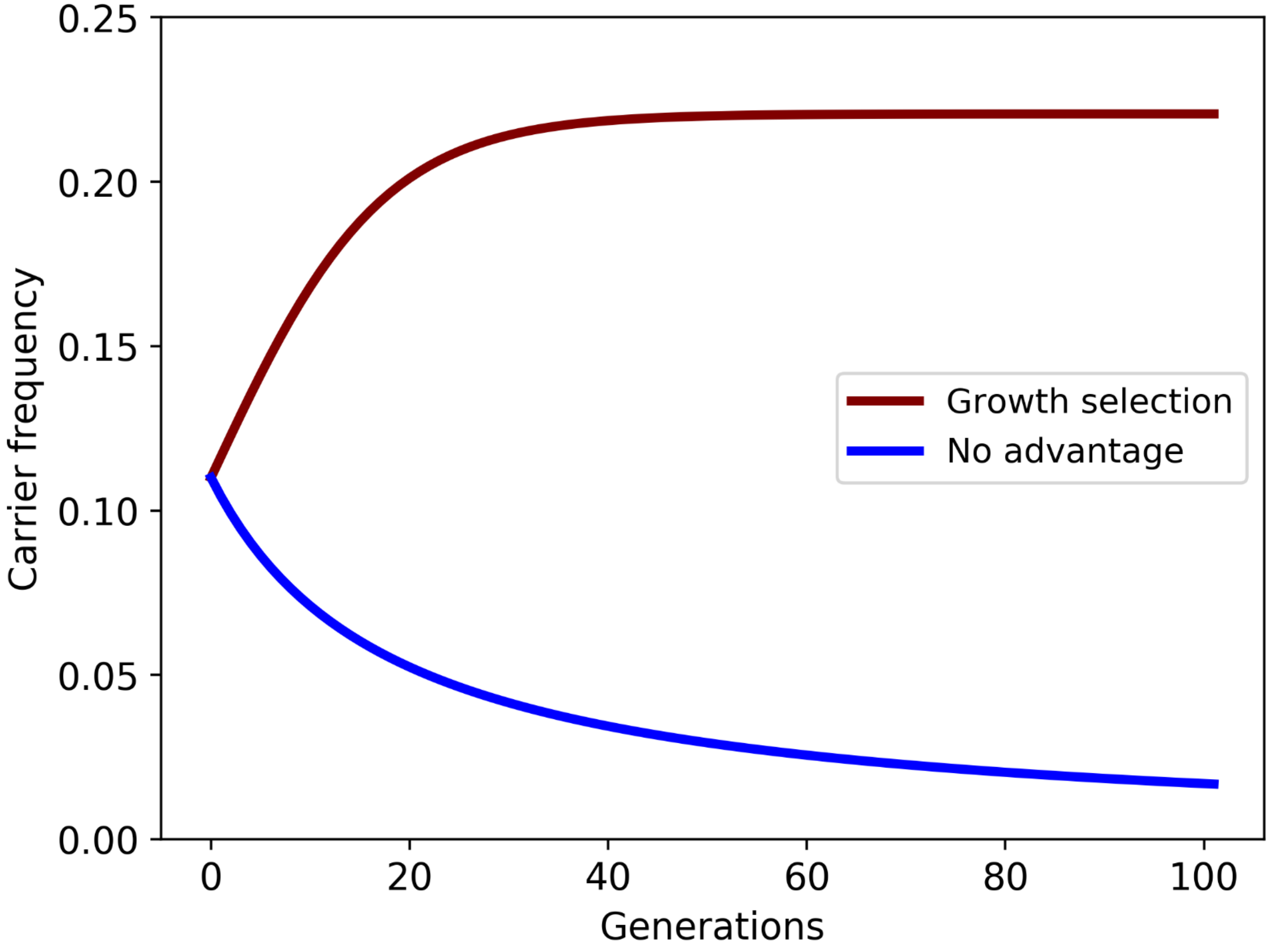

Figure S10: Simulation of the SSC18 carrier frequency if selection would be applied exclusively on growth. Figure shows fast increase in carrier frequency due to selective advantage on growth for carriers (12.4%).
