# Supplementary material for: Balancing selection on a recessive lethal deletion with pleiotropic effects on two neighboring genes in the porcine genome
Source: PLoS Genet. 2018 Sep 19;14(9):e1007661. doi: 10.1371/journal.pgen.1007661 (PMC6166978; doi:10.1371/journal.pgen.1007661)
Supplement: S1 Table — (PDF) [file pgen.1007661.s011.pdf]

Tables

Table S1: Results from two CxC (CC1: 11-may-2017, CC3: 19-may-2017) matings on farm 1. All mummified piglets were measured and sampled for genotyping, but not stored.

Litter: CC1	Class	Birth weight	Length (mm)	Approximate age (days)	Calls	Call rate	SSC18 status
2847	Liveborn	1360			50142	0.989	Carrier
2848	Liveborn	1260			50195	0.990	Carrier
2849	Liveborn	1620			50157	0.989	Non-carrier
2850	Liveborn	1100			50157	0.989	Carrier
2851	Liveborn	1600			50101	0.988	Carrier
2853	Liveborn	1380			50183	0.990	Non-carrier
2846	Stillborn	1220			49974	0.986	Carrier
2855	Mummified		140	60-70	35618	0.702	NA (Insufficient call rate)
2856	Mummified		110	50-60	31054	0.612	NA (Insufficient call rate)
2857	Mummified		160	60-70	35200	0.694	NA (Insufficient call rate)
2858	Mummified		150	60-70	32294	0.637	NA (Insufficient call rate)
**Litter: CC3**							
2912	Liveborn	1120			50121	0.989	Non-carrier
2913	Liveborn	1540			50132	0.989	Carrier
2914	Liveborn	960			50179	0.990	Non-carrier
2915	Liveborn	1600			50102	0.988	Non-carrier
2916	Liveborn	1180			50138	0.989	Carrier
2917	Liveborn	1800			50147	0.989	Carrier
2918	Liveborn	1320			50146	0.989	Carrier
2919	Liveborn	1300			50047	0.987	Carrier
2920	Liveborn	1040			50151	0.989	Homozygous
2921	Liveborn	1080			50162	0.989	Non-carrier
2923	Liveborn	980			50152	0.989	Non-carrier
2924	Liveborn	1520			50155	0.989	Carrier
2925	Liveborn	700			50188	0.990	Carrier
2927	Liveborn	740			50092	0.988	Non-carrier
2928	Liveborn	1350			50102	0.988	Carrier
2929	Stillborn	1340			50190	0.990	Non-carrier
2930	Stillborn	1410			50047	0.987	Carrier
2931	Stillborn	1310			50129	0.989	Homozygous
2932	Mummified		120	50-60	30136	0.594	NA (Insufficient call rate)
2933	Mummified		150	60-70	33254	0.656	NA (Insufficient call rate)
