# Supplementary material for: Balancing selection on a recessive lethal deletion with pleiotropic effects on two neighboring genes in the porcine genome
Source: PLoS Genet. 2018 Sep 19;14(9):e1007661. doi: 10.1371/journal.pgen.1007661 (PMC6166978; doi:10.1371/journal.pgen.1007661)
Supplement: S2 Table — (PDF) [file pgen.1007661.s012.pdf]

Table S2: Results from two CxC (CC2: 18-may-2017 , CC4: 01-jul-2017) matings on farm 2. All mummified piglets were sampled, measured and stored.

Litter: CC2	Class	Sex	Birth weight	NR teats	Length (mm)	Approximate age (days)	Calls	Call rate	SSC18 status
7347	Liveborn	Z	1390	17	-	-	50266	0.991	Carrier
7348	Liveborn	Z	1190	17	-	-	50282	0.992	Carrier
7349	Liveborn	Z	1330	16	-	-	50262	0.991	Carrier
7350	Liveborn	Z	1270	16	-	-	50263	0.991	Non-carrier
7351	Liveborn	Z	1410	17	-	-	50280	0.992	Carrier
7352	Liveborn	Z	1350	17	-	-	50238	0.991	Non-carrier
7353	Liveborn	Z	1200	16	-	-	50263	0.991	Carrier
7354	Liveborn	Z	1280	17	-	-	50270	0.991	Carrier
7355	Liveborn	Z	1100	14	-	-	50275	0.992	Carrier
7356	Liveborn	Z	1210	16	-	-	50284	0.992	Carrier
7357	Liveborn	Z	1150	17	-	-	50276	0.992	Non-carrier
7358	Liveborn	B	1150	16	-	-	-	-	-
7359	Mummified	-	156		150	60-70	31326	0.618	NA (Insufficient call rate)
7360	Mummified	-	149		180	70-80	43734	0.863	Homozygous
7361	Mummified	-	45		100	50-60	30228	0.596	NA (Insufficient call rate)
**Litter: CC4**									
8594	Liveborn	Z	1690	16			50280	0.992	Carrier
8595	Liveborn	Z	1920	17			50281	0.992	Carrier
8596	Liveborn	Z	1780	16			50263	0.991	Carrier
8597	Liveborn	B	1110	16			50283	0.992	Carrier
8598	Liveborn	B	1890	17			50275	0.992	Non-carrier
8599	Liveborn	B	1690	15			50278	0.992	Carrier
8600	Liveborn	B	1570	17			50284	0.992	Non-carrier
8601	Liveborn	B	1010	17			50286	0.992	Non-carrier
8602	Liveborn	B	1240	16			50291	0.992	Non-carrier
8603	Liveborn	B	1200	11			50293	0.992	Carrier
8604	Stillborn	Z	1370	-			50281	0.992	Carrier
8605	Stillborn	Z	1440	-			50282	0.992	Carrier
8606	Stillborn	Z	1290	-			34105	0.673	NA (Insufficient call rate)
8607	Mummified	-	520	-	230	~ 90	29854	0.589	NA (Insufficient call rate)
8608	Mummified	-	710	-	270	100-105	33050	0.652	NA (Insufficient call rate)
8609	Mummified	-	190	-	170	70-75	41119	0.811	Homozygous
8610	Mummified	-	130	-	150	60-70	29380	0.579	NA (Insufficient call rate)
8612	Mummified	-	640	-	240	90-95	29757	0.587	NA (Insufficient call rate)
