# Supplementary material for: Balancing selection on a recessive lethal deletion with pleiotropic effects on two neighboring genes in the porcine genome
Source: PLoS Genet. 2018 Sep 19;14(9):e1007661. doi: 10.1371/journal.pgen.1007661 (PMC6166978; doi:10.1371/journal.pgen.1007661)
Supplement: S5 Table — (PDF) [file pgen.1007661.s015.pdf]

Table S5: WGS sequenced individuals in the Large White breed.

SampleID	Coverage	SSC18 status	#Reads	#Sequenced bases	Read length	% Mapped
PigWUR166	22.674	carrier	608,750,200	57,222,518,800	100	99.24%
LW22F01	6.6232	non-carrier	177,936,643	16,562,211,035	101	99.59%
LW22F02	9.9578	carrier	267,856,550	24,899,229,748	101	99.43%
LW22F03	9.8968	carrier	264,911,155	24,746,926,946	101	99.46%
LW22F04	9.96	carrier	265,669,154	24,904,096,754	101	98.13%
LW22F05	9.6347	carrier	257,722,939	24,079,693,604	101	97.85%
LW22F06	9.3295	non-carrier	248,731,667	23,328,526,350	101	98.05%
LW22F07	9.1335	carrier	252,605,085	22,837,530,123	101	97.55%
LW22F08	10.3408	carrier	268,690,146	25,856,108,136	101	99.40%
LW22F09	12.1413	carrier	314,774,576	30,357,966,260	101	99.44%
LW22M04	9.5338	non-carrier	263,205,991	23,838,752,468	101	99.64%
LW22M07	12.7877	non-carrier	359,002,792	31,974,815,127	101	98.93%
LW39M01	12.0456	non-carrier	308,659,321	30,118,256,064	101	99.51%
LW39M04	9.8496	non-carrier	256,384,809	24,629,013,382	101	99.14%
LW39M05	12.4331	non-carrier	321,902,613	31,087,750,169	101	99.49%
LW39M07	11.6265	non-carrier	300,929,036	29,070,471,715	101	99.45%
Pig_271	8.6811	non-carrier	162,666,743	21,700,763,987	150	95.30%
Pig_280	9.0739	non-carrier	164,486,707	22,682,307,392	150	98.17%
Pig_281	9.1411	non-carrier	164,879,160	22,851,204,472	150	99.03%
Pig_285	9.2091	non-carrier	166,000,653	23,019,344,155	150	95.68%
Pig_286	9.5185	non-carrier	168,576,764	23,792,388,170	150	97.78%
Pig_287	8.0307	non-carrier	160,425,079	20,073,092,779	150	86.68%
Pig_288	9.1141	non-carrier	163,581,581	22,781,481,230	150	96.13%
Pig_289	9.4107	non-carrier	167,096,077	23,524,181,055	150	98.37%
Pig_290	8.9508	non-carrier	169,701,942	22,374,842,762	150	91.45%
Pig_291	9.6678	non-carrier	171,941,667	24,166,425,826	150	98.32%
Pig_292	9.921	non-carrier	179,801,757	24,798,270,362	150	97.16%
Pig_293	9.1906	non-carrier	179,571,591	22,972,645,138	150	91.87%
Pig_294	9.3796	non-carrier	167,928,810	23,445,890,822	150	97.26%
Pig_295	9.6204	non-carrier	173,548,973	24,046,997,276	150	96.53%
Pig_296	9.0105	carrier	171,403,077	22,524,223,410	150	91.73%
Pig_297	9.6456	non-carrier	170,065,207	24,111,212,748	150	99.09%
Pig_298	9.8891	non-carrier	177,346,139	24,720,329,598	150	97.71%
Pig_299	10.2072	non-carrier	185,749,725	25,515,612,767	150	96.48%
Pig_300	9.7504	non-carrier	173,668,789	24,374,120,618	150	98.47%
Pig_301	9.4642	non-carrier	168,169,962	23,658,511,841	150	98.94%
Pig_302	9.2307	non-carrier	165,493,884	23,075,342,621	150	97.68%
Pig_303	9.5695	non-carrier	172,825,217	23,921,741,267	150	96.75%
Pig_304	9.7225	non-carrier	175,941,522	24,304,289,703	150	96.90%
Pig_305	9.524	non-carrier	172,969,524	23,807,147,874	150	96.33%
Pig_306	9.3734	non-carrier	173,290,024	23,431,185,342	150	94.76%
Pig_307	9.6482	non-carrier	172,273,441	24,118,139,411	150	98.48%
Pig_308	9.7989	non-carrier	174,234,588	24,494,269,215	150	98.47%
Pig_309	9.2527	non-carrier	166,380,344	23,128,606,445	150	97.37%
Pig_312	10.1746	non-carrier	194,154,540	25,435,094,514	150	92.13%
Pig_320	9.3486	non-carrier	168,742,805	23,368,198,772	150	98.59%
Pig_321	9.4375	non-carrier	167,167,511	23,591,163,650	150	99.30%
Pig_322	9.26	non-carrier	163,560,192	23,147,366,012	150	99.31%
Pig_323	9.1623	non-carrier	164,679,080	22,902,924,044	150	98.76%
Pig_324	9.3366	non-carrier	165,369,259	23,339,342,226	150	99.15%
Pig_325	9.2747	non-carrier	166,146,180	23,184,714,748	150	98.83%
Pig_326	9.1765	non-carrier	164,375,336	22,938,829,458	150	99.12%
Pig_327	9.4078	non-carrier	176,306,083	23,513,414,676	150	98.67%
Pig_328	8.856	carrier	162,974,945	22,138,210,607	150	96.01%
Pig_329	9.1047	non-carrier	164,042,533	22,758,271,132	150	99.15%
Pig_330	9.1563	non-carrier	163,406,635	22,888,129,147	150	99.07%
PigWUR189	7.0507	non-carrier	295,973,118	17,629,617,414	100	60.94%
PigWUR190	6.6017	non-carrier	291,768,839	16,506,744,192	100	57.87%

PigWUR191	6.9729	non-carrier	298,219,605	17,435,220,241	100	59.94%
PigWUR192	11.4379	non-carrier	295,159,251	28,600,086,223	100	99.45%
PigWUR193	11.4612	non-carrier	301,523,521	28,658,289,443	100	97.82%
PigWUR194	11.5268	non-carrier	297,162,740	28,821,596,745	100	99.41%
PigWUR195	10.0891	non-carrier	297,040,954	25,226,940,395	100	87.11%
PigWUR196	11.2783	non-carrier	292,871,246	28,200,412,979	100	99.32%
PigWUR198	11.1232	non-carrier	301,469,201	27,812,503,597	100	99.20%
PIGWUR-256	11.2264	non-carrier	240,044,048	28,066,125,699	125	99.28%
PIGWUR-257	11.2581	non-carrier	241,827,827	28,145,871,295	125	99.16%
PIGWUR-258	11.2366	non-carrier	238,929,804	28,092,421,711	125	99.30%
PIGWUR-259	11.1662	non-carrier	240,422,405	27,915,921,966	125	99.17%
PIGWUR-260	11.2008	non-carrier	241,400,841	28,002,395,443	125	99.16%
PIGWUR-261	11.2736	non-carrier	245,049,225	28,184,548,554	125	99.07%
PIGWUR-262	11.3413	non-carrier	246,879,976	28,353,405,448	125	99.03%
PIGWUR-263	11.3816	non-carrier	253,939,030	28,454,078,052	125	99.30%
