# Supplementary material for: Balancing selection on a recessive lethal deletion with pleiotropic effects on two neighboring genes in the porcine genome
Source: PLoS Genet. 2018 Sep 19;14(9):e1007661. doi: 10.1371/journal.pgen.1007661 (PMC6166978; doi:10.1371/journal.pgen.1007661)
Supplement: S6 Table — (PDF) [file pgen.1007661.s016.pdf]

Table S6: Gene expression measured in fragments per kilobase per million (FPKM) for *BBS9* and *BMPER* gene in one SSC18 deletion carrier animal.

Tissue	Brain	Liver	Muscle	Spleen	Testis	Lung	Lymph	Olf bulb
**BMPER**	5.60	5.19	2.03	2.90	5.71	35.44	9.58	6.95
**BBS9**	4.19	1.90	0.482	4.14	7.95	4.95	5.91	5.36
