# Supplementary material for: Balancing selection on a recessive lethal deletion with pleiotropic effects on two neighboring genes in the porcine genome
Source: PLoS Genet. 2018 Sep 19;14(9):e1007661. doi: 10.1371/journal.pgen.1007661 (PMC6166978; doi:10.1371/journal.pgen.1007661)
Supplement: S7 Table — Heterozygous coding variants of the BMPER canonical transcript (XM_013990842.2) are used to test for allelic imbalance. (PDF) [file pgen.1007661.s017.pdf]

Table S7: Allele specific expression test of the *BMPER* gene for three non-carriers pigs. Heterozygous coding variants of the *BMPER* canonical transcript (XM_013990842.2) are used to test for allelic imbalance. The LW non-carrier pig RNA-seq data was downloaded from Li et al 2017 (1).

Sample	Tissue	Locus	Gene	Ref	Alt	Ref- Count	Alt- Count	Ratio
LW	Lung	18: 39598668	*BMPER*	C	A	36	46	0.561
PI	Lung	18: 39594479	*BMPER*	C	T	40	55	0.579
DU	Testis	18: 39594479	*BMPER*	C	T	6	6	0.5

References

Li MZ, Chen L, Tian SL, Lin Y, Tang QZ, Zhou XM, et al. Comprehensive variation discovery and recovery of missing sequence in the pig genome using multiple de novo assemblies. Genome research. 2017;27(5):865-74.
