# Supplementary material for: Balancing selection on a recessive lethal deletion with pleiotropic effects on two neighboring genes in the porcine genome
Source: PLoS Genet. 2018 Sep 19;14(9):e1007661. doi: 10.1371/journal.pgen.1007661 (PMC6166978; doi:10.1371/journal.pgen.1007661)
Supplement: S8 Table — (PDF) [file pgen.1007661.s018.pdf]

Table S8: Deletion carrier frequency and the number of genotyped animals per time point from 2006 - 2018.

Year	Month	#Carriers	#Non-carriers	#Total genotyped	Carrier frequency
2006	7	6	32	38	0.158
2007	1	11	70	81	0.136
2007	7	29	170	199	0.146
2008	1	56	264	320	0.175
2008	7	118	468	586	0.201
2009	1	142	611	753	0.189
2009	7	139	681	820	0.17
2010	1	155	778	933	0.166
2010	7	170	918	1088	0.156
2011	1	175	1139	1314	0.133
2011	7	168	1241	1409	0.119
2012	1	172	1472	1644	0.105
2012	7	189	1638	1827	0.103
2013	1	189	1693	1882	0.1
2013	7	204	1798	2002	0.102
2014	1	217	1957	2174	0.1
2014	7	289	2546	2835	0.102
2015	1	449	4318	4767	0.094
2015	7	513	5652	6165	0.083
2016	1	594	5415	6009	0.099
2016	7	758	6524	7282	0.104
2017	1	887	6673	7560	0.117
2017	7	852	6778	7630	0.112
2018	1	481	3574	4055	0.119
