# Supplementary material for: Balancing selection on a recessive lethal deletion with pleiotropic effects on two neighboring genes in the porcine genome
Source: PLoS Genet. 2018 Sep 19;14(9):e1007661. doi: 10.1371/journal.pgen.1007661 (PMC6166978; doi:10.1371/journal.pgen.1007661)
Supplement: S9 Table — (PDF) [file pgen.1007661.s019.pdf]

Table S9: Dumeco-W carriers for SSC18 deletion including birth dates. The final column shows the average LRR signal intensity for the four overlapping deletion makers.

Animal ID	Line	Birth-date (dd-mm-yyyy)	Average LRR signal intensity
02A69	Dumeco - W	23-05-2001	-0.435
03A04	Dumeco - W	24-05-2001	-0.426
03A08	Dumeco - W	24-05-2001	-0.359
4367DDVB	Dumeco - W	30-04-2000	-0.423
6479DDVB	Dumeco - W	23-09-2000	-0.405
