# Supplementary material for: Balancing selection on a recessive lethal deletion with pleiotropic effects on two neighboring genes in the porcine genome
Source: PLoS Genet. 2018 Sep 19;14(9):e1007661. doi: 10.1371/journal.pgen.1007661 (PMC6166978; doi:10.1371/journal.pgen.1007661)
Supplement: S10 Table — (PDF) [file pgen.1007661.s020.pdf]

Table S10: Association analysis using deregressed breeding values (DEBV) for 16 traits in the Large White breed. Effect shows the direction of the association, se shows the standard error. The number of carriers and non-carriers can differ per trait depending on the reliability of the DEBVs. Green colour indicates positively affected traits, red indicates negatively affected traits in the breeding program.

TRAIT (unit)	Non-carriers	Carriers	P	-log10(P)	effect	se
Test growth rate (gr/day)	15013	1605	0.000046	4.34	11.46	2.81
Loin depth (mm)	15011	1598	0.000198	3.70	-0.45	0.12
Lifetime growth rate	15116	1616	0.000315	3.50	6.40	1.77
Litter birth weight (gram)	6945	824	0.001232	2.91	-16.67	5.16
Litter mortality (%)	7345	871	0.001248	2.90	0.67	0.21
Daily feed intake (gr/day)	14671	1567	0.006764	2.17	30.61	11.30
Longevity (parity)	7250	856	0.024828	1.61	-0.08	0.04
Stillbirths (number)	7574	890	0.197659	0.70	-0.03	0.03
Farrowing survival (%)	7125	835	0.292163	0.53	-0.19	0.18
Lactation survival (%)	7347	863	0.454298	0.34	0.20	0.27
Farrowing rate (%)	4840	545	0.610177	0.21	-0.17	0.34
Number of teats (number)	15804	1684	0.631532	0.20	0.01	0.03
Litter variation (gram)	6793	795	0.646788	0.19	0.81	1.76
Backfat (mm)	15144	1613	0.718438	0.14	0.02	0.06
Total number born	7468	879	0.862497	0.06	-0.01	0.07
Mothering ability (%)	7313	859	0.920348	0.04	0.01	0.16
