# Supplementary material for: Balancing selection on a recessive lethal deletion with pleiotropic effects on two neighboring genes in the porcine genome
Source: PLoS Genet. 2018 Sep 19;14(9):e1007661. doi: 10.1371/journal.pgen.1007661 (PMC6166978; doi:10.1371/journal.pgen.1007661)
Supplement: S11 Table — (PDF) [file pgen.1007661.s021.pdf]

Table S11: Estimated Breeding Values (EBV) for seven traits including the overall selection index (TSI). Table shows EBVs for all animals born in 2017, divided in two classes (C=carriers, NC=non-carriers). Carriers have greater TSI and growth EBV, while lower EBV for daily feed intake.

EBVs	TSI	Growth rate	Daily feed intake	Total number born	Stillbirths	Litter mortality	Vitality
C (n=412)	112.130	19.618	-10.095	0.009	-0.204	-1.256	1.242
NC (n=3030)	109.240	17.450	-4.968	-0.043	-0.133	-1.137	0.900
Ratios	1.027	1.124	2.032	-0.207	1.535	1.104	1.380
