# Supplementary material for: Balancing selection on a recessive lethal deletion with pleiotropic effects on two neighboring genes in the porcine genome
Source: PLoS Genet. 2018 Sep 19;14(9):e1007661. doi: 10.1371/journal.pgen.1007661 (PMC6166978; doi:10.1371/journal.pgen.1007661)
Supplement: S12 Table — (PDF) [file pgen.1007661.s022.pdf]

Table S12: Primer information for the genes used for RT-qPCR.

Gene	Forward primer	Reverse primer	Exon junction
GAPDH	5′-AGTATGATTCCACCCACGGC-3′	5′-GGATCTCGCTCCTGGAAGATG-3′	Exon 4-5
BBS9	5′-TCATGCCAAGCACAGACGAA-3′	5′-TCTGCTGCATTTCCTAGTGTC-3′	Exon 23-24
