# Supplementary material for: Balancing selection on a recessive lethal deletion with pleiotropic effects on two neighboring genes in the porcine genome
Source: PLoS Genet. 2018 Sep 19;14(9):e1007661. doi: 10.1371/journal.pgen.1007661 (PMC6166978; doi:10.1371/journal.pgen.1007661)
Supplement: S13 Table — (PDF) [file pgen.1007661.s023.pdf]

Table S13: RT-qPCR results for *BBS9* expression in 8 carriers and 10 non-carriers.

ID	Status	mean Ct BBS9	mean Ct GAPDH	Δ Ct (BBS9/GADPH)	ΔΔ Ct (BBS9/GADPH)	Expression fold change
Sample1	carrier	28.473	20.225	8.2	0.1	0.9
Sample2	carrier	28.973	20.580	8.4	0.3	0.8
Sample3	carrier	29.497	20.476	9.0	0.9	0.5
Sample4	carrier	29.794	21.006	8.8	0.7	0.6
Sample5	carrier	28.734	20.046	8.7	0.6	0.7
Sample6	carrier	29.971	21.300	8.7	0.5	0.7
Sample7	carrier	29.447	20.974	8.5	0.3	0.8
Sample8	carrier	28.783	19.840	8.9	0.8	0.6
Sample9	non_carrier	27.646	20.409	7.2	-0.9	1.9
Sample10	non_carrier	27.981	20.714	7.3	-0.9	1.8
Sample11	non_carrier	29.546	21.524	8.0	-0.1	1.1
Sample12	non_carrier	29.248	21.018	8.2	0.1	0.9
Sample13	non_carrier	27.575	19.786	7.8	-0.3	1.3
Sample14	non_carrier	27.510	20.932	6.6	-1.6	2.9
Sample15	non_carrier	31.347	23.391	8.0	-0.2	1.1
Sample16	non_carrier	27.111	19.290	7.8	-0.3	1.2
Sample17	non_carrier	28.940	20.781	8.2	0.0	1.0
Sample18	non_carrier	28.235	20.195	8.0	-0.1	1.1
